# Structural Basis of Response Regulator Dephosphorylation by Rap Phosphatases

**DOI:** 10.1371/journal.pbio.1000589

**Published:** 2011-02-08

**Authors:** Vijay Parashar, Nicolas Mirouze, David A. Dubnau, Matthew B. Neiditch

**Affiliations:** 1 Department of Microbiology and Molecular Genetics, UMDNJ–New Jersey Medical School, Newark, New Jersey, United States of America; 2 Public Health Research Institute Center, UMDNJ–New Jersey Medical School, Newark, New Jersey, United States of America; University of North Carolina, United States of America

## Abstract

Bacterial Rap family proteins have been most extensively studied in *Bacillus subtilis*, where they regulate activities including sporulation, genetic competence, antibiotic expression, and the movement of the ICEBs1 transposon. One subset of Rap proteins consists of phosphatases that control *B. subtilis* and *B. anthracis* sporulation by dephosphorylating the response regulator Spo0F. The mechanistic basis of Rap phosphatase activity was unknown. Here we present the RapH-Spo0F X-ray crystal structure, which shows that Rap proteins consist of a 3-helix bundle and a tetratricopeptide repeat domain. Extensive biochemical and genetic functional studies reveal the importance of the observed RapH-Spo0F interactions, including the catalytic role of a glutamine in the RapH 3-helix bundle that inserts into the Spo0F active site. We show that in addition to dephosphorylating Spo0F, RapH can antagonize sporulation by sterically blocking phosphoryl transfer to and from Spo0F. Our structure-function analysis of the RapH-Spo0F interaction identified Rap protein residues critical for Spo0F phosphatase activity. This information enabled us to assign Spo0F phosphatase activity to a Rap protein based on sequence alone, which was not previously possible. Finally, as the ultimate test of our newfound understanding of the structural requirements for Rap phosphatase function, a non-phosphatase Rap protein that inhibits the binding of the response regulator ComA to DNA was rationally engineered to dephosphorylate Spo0F. In addition to revealing the mechanistic basis of response regulator dephosphorylation by Rap proteins, our studies support the previously proposed T-loop-Y allostery model of receiver domain regulation that restricts the aromatic “switch” residue to an internal position when the β4-α4 loop adopts an active-site proximal conformation.

## Abstract 2

Crystallographic, biochemical, and genetic studies reveal the mechanism of Rap protein phosphatase activity within the phosphorelay pathway leading to sporulation in *Bacillus* species.

## Author Summary

A phosphorelay signal transduction pathway regulates sporulation in numerous *Bacillus* species including the genetic model organism, *B. subtilis*, and the causative agent of anthrax, *B. anthracis*. Histidine kinases initiate the flow of phosphoryl groups along the phosphorelay pathway, which then shuttles them to a downstream response-regulator transcription factor called Spo0A. Ultimately, sporulation is governed by the cellular concentration of phosphorylated Spo0A. In numerous *Bacillus* species, Rap phosphatases function in opposition to the histidine kinases, inhibiting Spo0A activation by dephosphorylating an intermediate pathway protein called Spo0F. Here we present the structure of a Rap protein, RapH, in complex with Spo0F, as determined by X-ray crystallography. The RapH–Spo0F structure, along with biochemical and genetic studies, reveals the mechanism of Rap-protein-mediated Spo0F dephosphorylation. We used information gleaned from our structure–function analysis, first, to assign Spo0F phosphatase activity to an uncharacterized Rap protein, RapJ, on the basis of sequence alone, and, second, to engineer Spo0F phosphatase activity de novo into a non-phosphatase Rap protein, RapF. We found that in addition to dephosphorylating Spo0F, Rap proteins can inhibit the sporulation phosphorelay by sterically blocking the transfer of phosphoryl groups to and from Spo0F. Ultimately, new classes of drugs might be developed that disrupt the flow of phosphoryl groups along phosphotransfer signaling pathways by mimicking the antagonistic effects of Rap proteins on response regulators.

## Introduction

**Figure 1:**
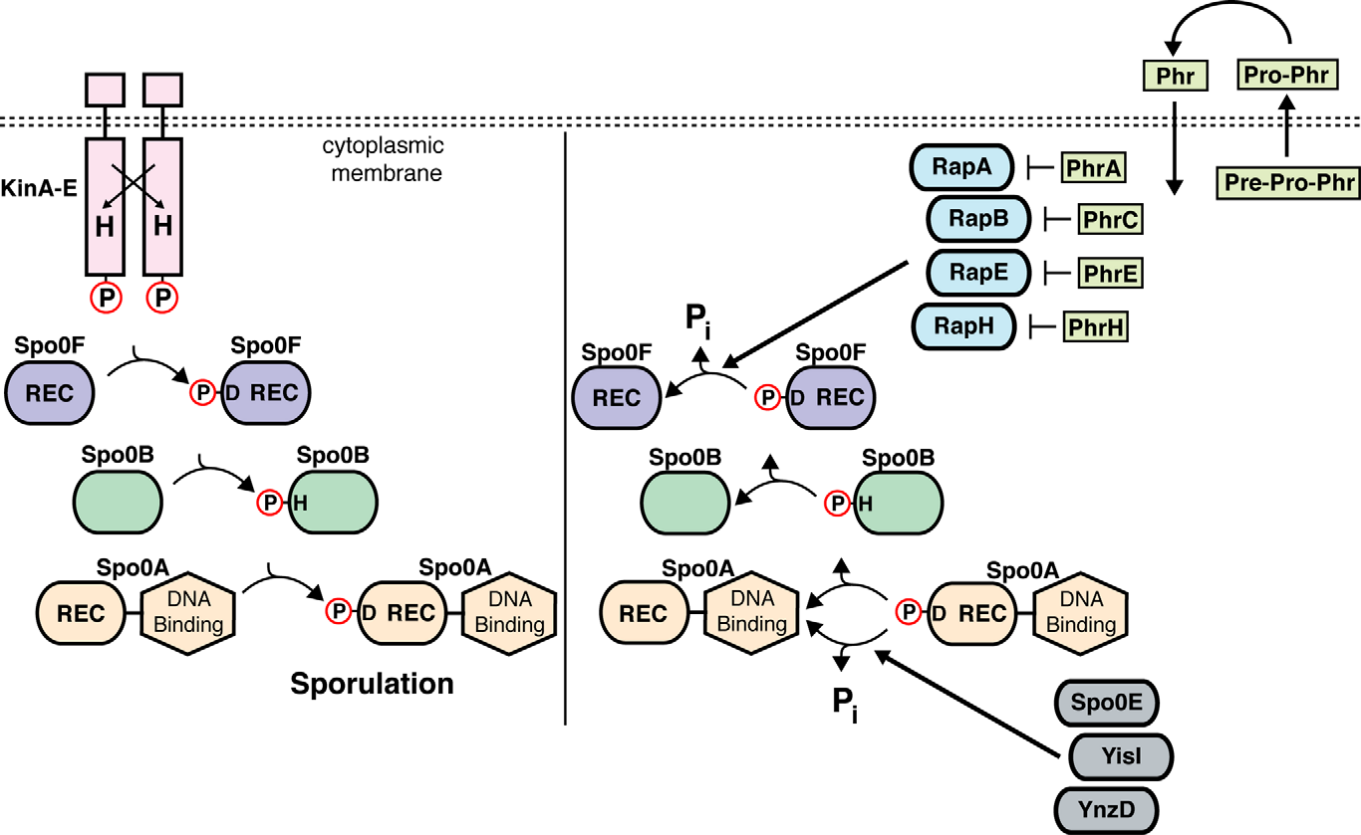
Rap proteins and Phr peptides regulate *B. subtilis* sporulation signal transduction. The *B. subtilis* sporulation kinases (KinA-E) are depicted as membranous proteins for the sake of simplicity; however, KinA and KinE are actually soluble cytoplasmic receptors. Phosphoryl groups are transferred to Spo0F, Spo0B, and ultimately to Spo0A. When the level of Spo0A∼P reaches a critical level, sporulation is triggered (left panel). Rap proteins dephosphorylate Spo0F∼P causing phosphoryl groups to flow away from Spo0A, inhibiting sporulation (right panel). The Spo0K permease (not pictured) imports Phr peptides into the cytoplasm where they positively regulate sporulation by binding to Rap proteins and inhibiting Spo0F dephosphorylation. The Spo0E family member phosphatases, Spo0E, YisI, and YnzD, dephosphorylate *B. subtilis* Spo0A [48],[49]. REC, Receiver Domain; H, histidine; D, aspartic acid; P, phosphoryl group; P_i_, inorganic phosphate.

*B. subtilis* spore development is regulated by a phosphorelay signal transduction pathway that is the prototype for all phosphorelay signal transduction pathways in bacteria (Figure 1; [1]). The *B. subtilis* sporulation signal transduction pathway is initiated by five histidine kinases—KinA, KinB, KinC, KinD, and KinE—that autophosphorylate in response to unknown environmental and physiological cues coincident with nutritional starvation (Figure 1, left panel; [2]). Signaling converges on the centrally important intermediate response regulator Spo0F, which autophosphorylates using phosphohistidines in a sporulation histidine kinase as a phosphoryl donor. Spo0F then phosphorylates the histidine phosphotransfer (HPt) protein Spo0B, which in the final pathway step relays phosphoryl groups to Spo0A. Phosphorylated Spo0A then directly activates or represses 121 genes comprising the Spo0A regulon [3]. In total, however, at least 520 *B. subtilis* genes are significantly upregulated or downregulated as a result of Spo0A phosphorylation [4].

Phosphorelay pathway intermediate response regulator proteins like Spo0F, which consist entirely of a single receiver (REC) domain identical in structure to the N-terminal REC domains of transcription factor response regulators, dephosphorylate by transferring their phosphoryl groups to water in autohydrolysis reactions (Figure 1, right panel; [5],[6]). The dephosphorylation of intermediate response regulators causes the direction of phosphoryl flow to reverse (Figure 1, right panel). That is, the intermediate response regulator pulls phosphoryl groups from the HPt, which in turn dephosphorylates the downstream response regulator. The rate of response regulator dephosphorylation is determined by autohydrolysis activity intrinsic to the REC domain and the activity of response regulator-specific phosphatases, such as Rap proteins (Figure 1, right panel; [7],[8]).

Rap proteins, named after the founding members of the family, which were shown to be *r*esponse regulator *a*spartate *p*hosphatases, have been most thoroughly studied in *B. subtilis*, where they are central to the regulation of diverse developmental processes including sporulation and genetic competence [8],[9]. In *B. subtilis* alone there are 11 homologous Rap proteins encoded on the chromosome, and there are at least four additional Rap proteins encoded on *B. subtilis* plasmids [10],[11].

A subset of Rap proteins, consisting of RapA, RapB, RapE, and RapH, dephosphorylates Spo0F, consequently reducing the level of phosphorylated Spo0A in the cell and inhibiting sporulation (Figure 1, right panel; [8],[12],[13]).

We have determined the X-ray crystal structure of a Rap phosphatase, RapH, in complex with its target protein, Spo0F, which is bound to Mg^2+^, an important constituent of the Spo0F active site. The RapH-Spo0F crystallographic asymmetric unit contains RapH-Spo0F complexes in two different conformations, reflecting different steps along the Spo0F dephosphorylation pathway. To our knowledge, the RapH-Spo0F X-ray crystal structure represents the first structure of a Rap protein or a Rap protein in complex with its substrate. As expected from their amino acid sequences, Rap proteins have an overall structure that is radically different from the known structures of other bacterial phosphatases. The RapH-Spo0F crystal structure, along with in vitro biochemical and in vivo genetic data, provides mechanistic insight into Rap protein function, specifically the molecular basis of Rap phosphatase activity.

## Results

### Overall RapH-Spo0F Crystal Structure

**Figure 2:**
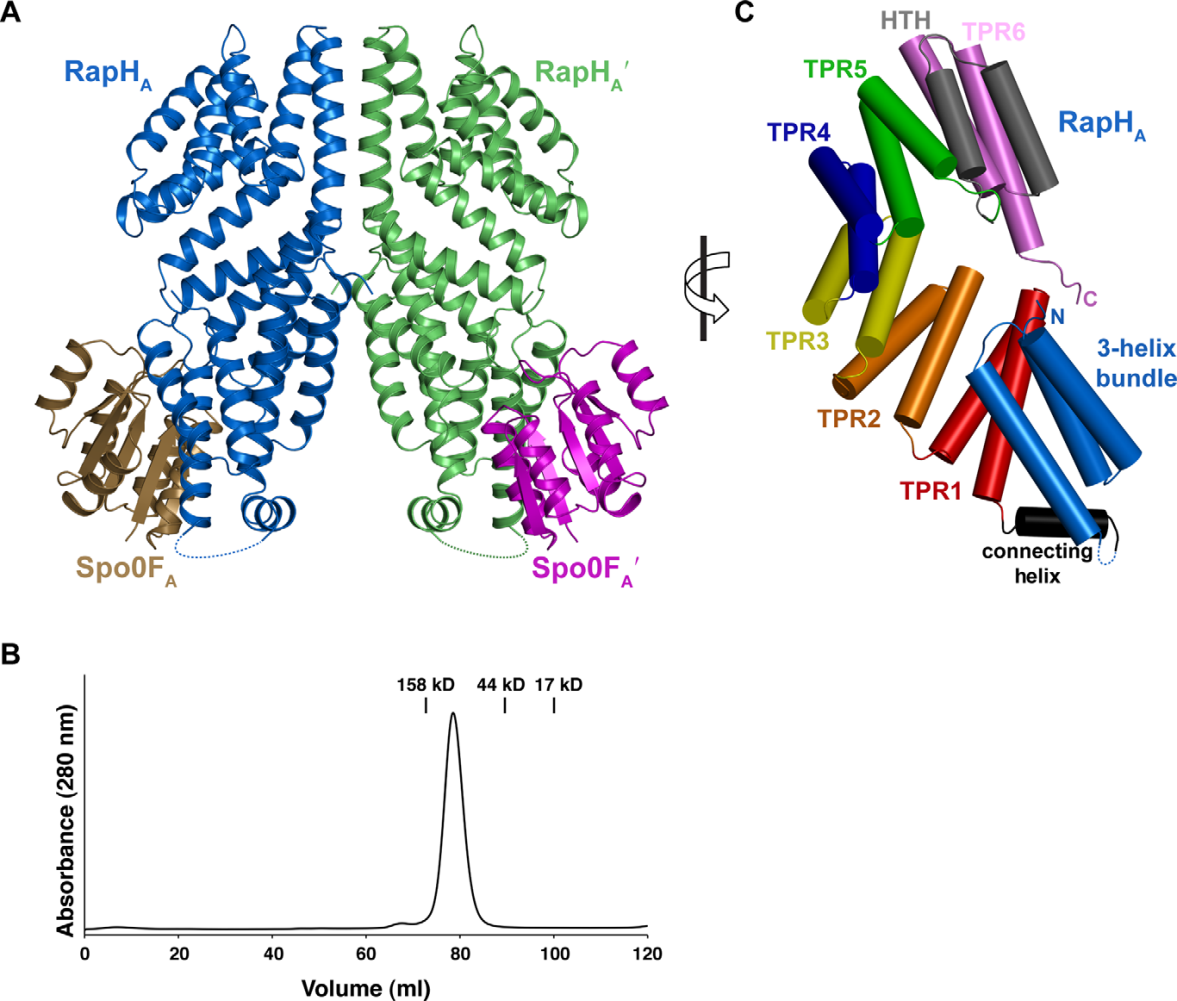
RapH-Spo0F structure. (A) The (RapH_A_-Spo0F_A_)_2_ heterotetramer comprised of RapH_A_ (blue), Spo0F_A_ (brown), RapH_A_' (green), and Spo0F_A_' (magenta). (B) Size exclusion chromatography shows that RapH (MW_theor_ 44.1 kDa) forms RapH_2_ dimers (MW_exper_ 85.1 kDa) in solution. The peak positions of gel filtration standards are indicted by vertical lines above the absorbance trace. (C) To obtain this view of RapH_A_, the structure illustrated in panel A was rotated 90° in the direction indicated by the arrow. The RapH_A_ N-terminal 3-helix bundle (light blue) is connected to the C-terminal TPR domain by a flexible linker (dashed lines) and a short helix (black cylinder). HTH, helix-turn-helix.

**Figure 3:**
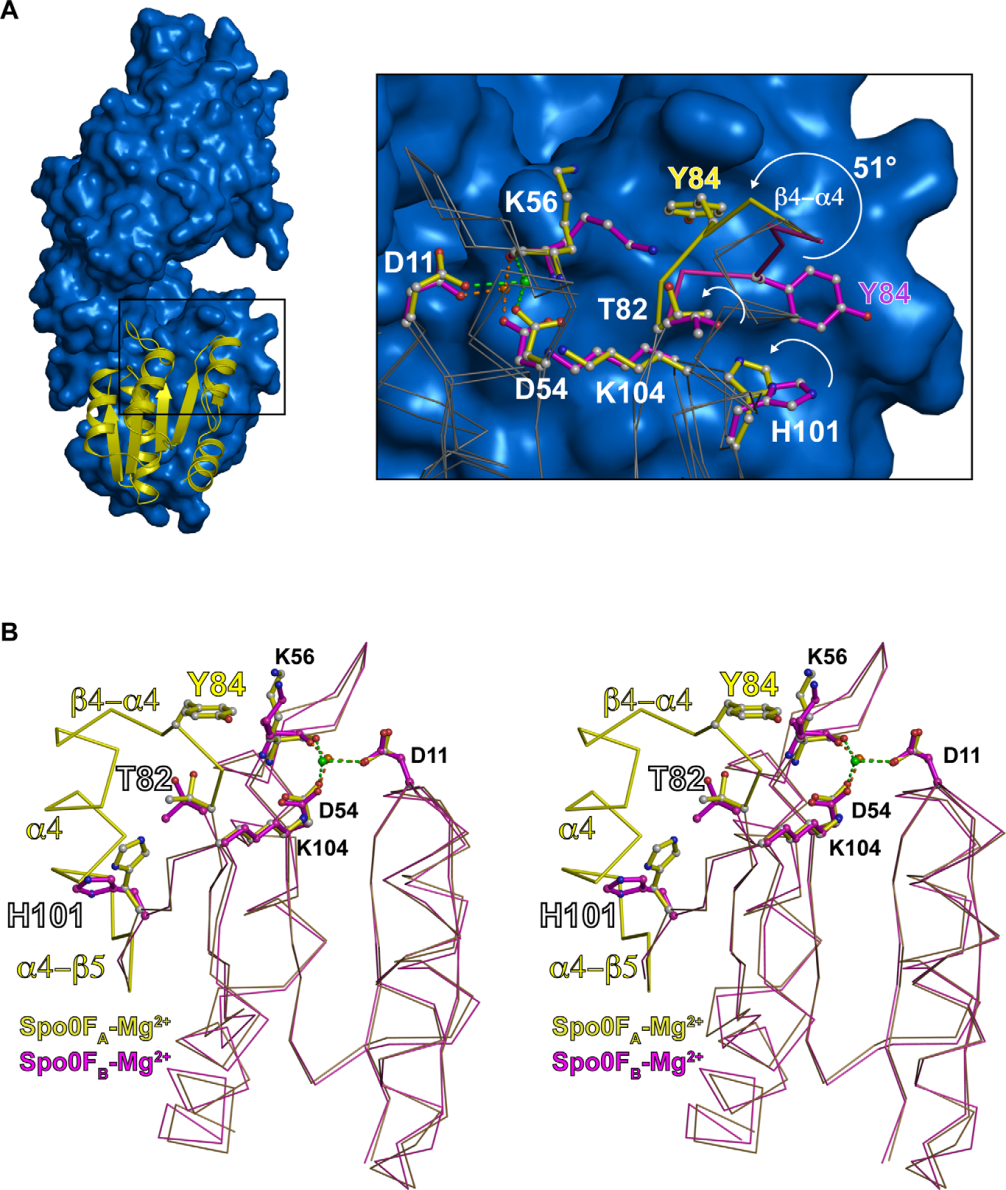
Spo0F structural alignments. (A, left panel) RapH_A_ (blue surface) in complex with Spo0F_A_-Mg^2+^ (yellow cartoon). (A, right panel) Expanded view of the area enclosed by the black rectangle in the left panel showing the structural alignment of Spo0F_A_-Mg^2+^ (grey ribbon with yellow sticks) and Spo0F-Mn^2+^ (grey ribbon with magenta sticks, PDB ID 1PEY). Mg^2+^ and Mn^2+^ are depicted as green and orange spheres, respectively. This alignment reveals that RapH_A_ causes Spo0F Thr82 to rotate towards the active site, Spo0F His101 to adopt an internalized conformation, and the Spo0F β4-α4 loop to shift towards the active site as indicated by the white curved arrows. Spo0F Lys104 could hydrogen bond with a phosphoryl group oxygen in the active-site phosphoaspartate (see Figure 9C). The pseudodihedral angle created by the Cα atoms equivalent to the Spo0F Cα atoms in residues 82, 83, 84, and 85 was previously used to quantify conformational changes in the FixJ and CheY receiver domain β4-α4 loops [34],[50],[51]. A rotation of 51° around this pseudodihedral angle is observed in the RapH_A_-Spo0F_A_ structure. The side chain of Spo0F_A_ Lys56 is disordered, thus avoiding a clash with the repositioned β4-α4 loop. (B) A stereoview depicting the structural alignment of Spo0F_A_ (yellow) and Spo0F_B_ (magenta) shows that Spo0F_A_ adopts a “phosphorylated” conformation and Spo0F_B_ adopts a “non-phosphorylated” conformation. The Spo0F_B_ β4-α4 loop, helix α4, and a portion of the α4-β5 loop are disordered. The Mg^2+^ ion coordinated by Spo0F_A_ Asp11, Asp54, and Lys56 is depicted by a green sphere. The Mg^2+^ ion coordinated by Spo0F_B_ Asp11, Asp54, and Lys56 is depicted by an orange sphere. Structural alignments were performed with DaliLite [45].

To determine the mechanistic basis of Rap phosphatase activity, we crystallized *B. subtilis* RapH in complex with its target response regulator Spo0F. The single wavelength anomalous diffraction method was used to obtain experimental phases and the RapH-Spo0F structure was ultimately refined to 2.20 Å resolution (Table S1). RapH forms dimers in the RapH-Spo0F crystals; this is consistent with gel filtration studies showing that RapH forms stable dimers in solution (Figures 2A, 2B, and S1A). Each RapH protomer in the RapH_2_ dimer is bound to a monomer of Spo0F, forming a (RapH-Spo0F)_2_ complex, and each Spo0F monomer coordinates a magnesium ion in its active site, consistent with the fact that magnesium was included in the protein purification, crystallization, and cryoprotection buffers (Figures 2A, 3A, and 3B).

As discussed in detail below, within single RapH-Spo0F crystals there are two RapH-Spo0F complexes in different conformations (Figures 2A, 2B, and S1A). To discuss the RapH-Spo0F structure, we have adopted the following nomenclature. The different RapH-Spo0F complexes in the crystallographic asymmetric unit are named RapH_A_-Spo0F_A_ and RapH_B_-Spo0F_B_ (Figures 2A and S1A). RapH_A_ dimerizes around a crystallographic 2-fold symmetry axis forming a (RapH_A_-Spo0F_A_)_2_ complex (Figure 2A). This complex is a heterotetramer comprised of RapH_A_, RapH_A_′, Spo0F_A_, and Spo0F_A_′, and it buries 4,127 Å^2^ of surface area at the large RapH_A_-RapH_A_′ interface. Similarly, RapH_B_ dimerizes around a crystallographic 2-fold symmetry axis forming (RapH_B_-Spo0F_B_)_2_ (Figure S1A). This heterotetramer is composed of RapH_B_, RapH_B_′, Spo0F_B_, and Spo0F_B_′, and it buries 4,187 Å^2^ surface area at the RapH_B_-RapH_B_′ interface. Where subscripts and superscripts are omitted from the protein names below, we refer to both of the non-identical copies of RapH or Spo0F in the crystallographic asymmetric unit.

### RapH Structure

**Figure 4:**
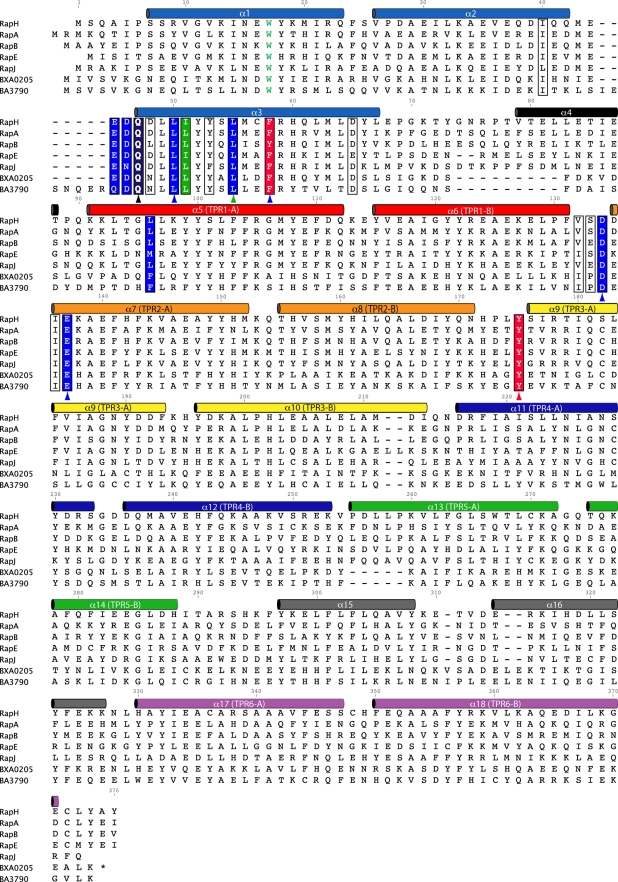
Rap phosphatase sequence alignment. The amino acid sequences of *Bacillus* Rap proteins previously demonstrated to dephosphorylate Spo0F in vitro were aligned using the Blosum62 matrix in Geneious Pro (Biomatters Ltd.). The residue numbers indicated above the sequences refer to RapH. The highly conserved tryptophan at the core of the N-terminal 3-helix bundle is colored green. The ConSurf server was used to determine surface residue conservation [46]. The highly conserved residues in the RapH-Spo0F interface are surrounded by black boxes. Alanine substitutions at the positions highlighted by red boxes resulted in the greatest loss of RapH function in vivo. Alanine substitutions at the positions highlighted by blue or green boxes resulted in intermediate or no loss of RapH function in vivo, respectively. Asparagine substitution at RapH residue Gln47, which is highlighted by a black box, resulted in an intermediate loss of RapH function in vivo. Alanine substitutions at the positions marked with red arrowheads resulted in the greatest loss of RapH function in vitro. Alanine substitutions at the positions marked with blue or green arrowheads resulted in intermediate or no loss of RapH function in vitro, respectively. Asparagine or glutamate substitution at RapH residue Gln47, which is marked with a black arrowhead and in bold type, resulted in complete loss-of-function or no loss-of-function in vitro, respectively. RapH secondary structure assignments were made using the PyMOL algorithm [41]. The secondary structure cylinder colors correspond to the coloring scheme used in Figures 2C and S1B.

The RapH-Spo0F X-ray crystal structure shows that RapH_A_ contains an N-terminal 3-helix bundle (α1-α3) (Figure 2A and 2C). RapH_B_ also appears to form an N-terminal 3-helix bundle, but in contrast to Spo0F_A_ the electron density corresponding to residues 27–42 of helix α2 and 43–45 of the α2-α3 loop were not clearly interpretable and these residues were not modeled (Figures S2, S1A, and S1B). Buried at the core of the RapH 3-helix bundle is tryptophan-17. Without exception, Rap proteins have tryptophan at this position, and an N-terminal 3-helix bundle is probably a universal feature of Rap proteins (Figure 4). The RapH-Spo0F structure suggests that the conserved tryptophan is important for the folding of the Rap protein N-terminal 3-helix bundle, and as discussed in detail below the Rap protein 3-helix bundle is centrally important for phosphatase function.

Connected to the C-terminus of the RapH 3-helix bundle via a flexible linker and a short helix (α4) is a large right-handed superhelical domain composed of seven pairs of antiparallel α-helices (Figures 2C and S1B). Each pair of antiparallel α-helices is a helix-turn-helix consisting of an N-terminal helix (helix A), a connecting loop, and a C-terminal helix (helix B) (Figure 4). The antiparallel α-helices comprising pairs 1–5 and pair 7 contain tetratricopeptide repeat (TPR) sequences which are 34 amino acid motifs frequently found in tandem (Figure 4). A pair of antiparallel α-helices (α15–α16) separating TPR5 and TPR6 resembles a TPR helix-turn-helix fold in its overall structure but does not encode the TPR motif signature residues [14]. Additionally, the loop (residues 289–296) connecting TPR5 helix B to helix α15 is longer than the other TPR fold-connecting loops, and residues 292–294 are disordered in RapH_B_ (Figures 4 and S1B). Because the RapH C-terminal domain consists of six bona fide TPR folds and a helix-turn-helix fold that largely resembles a TPR fold and contributes like a TPR fold to the C-terminal domain superhelix, we refer to the entire region (amino acids 77–376) as the TPR domain.

Overall, RapH_A_ and RapH_B_ have very similar tertiary structures and align with a root mean-square deviation of 0.9 Å for 332 Cα atoms modeled in both structures; however, there are very important conformational differences between them (see Figure S2). We attribute these differences largely to the distinct crystal packing environments of the RapH_A_ and RapH_B_ 3-helix bundles and α4 helices. For example, the RapH_A_ 3-helix bundle and helix α4 interact with the RapH_B_ TPR domain, burying 1,146.2 Å^2^ surface area (unpublished data). In contrast, residues in the RapH_B_ 3-helix bundle make far fewer crystallographic contacts, burying only 191.2 Å^2^ surface area. These differences may also explain why residues 27–42 of helix α2, 43–45 of the α2-α3 loop, and 77–85 of the region containing the connecting helix α4 are ordered in RapH_A_ but disordered in RapH_B_ (Figure S2). As described below, consistent with the conformational differences observed in RapH_A_ and RapH_B_, Spo0F_A_ and Spo0F_B_ are in different conformations.

### Spo0F Conformational Changes Caused by RapH

Divalent metal ions were previously shown to be required for both Rap-mediated Spo0F dephosphorylation as well as Spo0F autodephosphorylation [15],[16]. Consistent with these observations, both Spo0F_A_ and Spo0F_B_ are bound to Mg^2+^ (Figure 3B). Alignment of Spo0F_A_ with the previously determined crystal structure of Spo0F bound to Mn^2+^ (PDB ID 1PEY) revealed that RapH_A_ induces conformational changes in Spo0F_A_ (Figure 3A, right panel; [17]).

More specifically, this alignment showed that RapH_A_ binding causes the entire Spo0F_A_ β4-α4 loop to dramatically shift and flip toward the active site (Figure 3A, right panel). This conformational change is exemplified by Tyr84, positioned in the middle of the Spo0F_A_ β4-α4 loop, which experiences 2.1–5.0 Å displacements in its main-chain atoms and 5.4–10.3 Å shifts in its side-chain atoms (see Figure 3A, right panel). As described in detail below, the interaction of Spo0F_A_ Tyr84 with a conserved binding cleft in RapH_A_, formed by RapH_A_ residues Glu45, Asp46, and Leu50, appears to be critically important for RapH-mediated dephosphorylation of Spo0F, and alanine substituted for Tyr84 was previously shown to render Spo0F resistant to the phosphatase activity of the RapH homolog RapB [16].

RapH_A_ causes the Spo0F_A_ switch residue Thr82, located at the C-terminal end of strand β4, to translate approximately 1 Å and its sidechain to rotate more than 120 degrees towards the Spo0F_A_ active site (Figure 3A, right panel). The rotation of a conserved threonine or serine at this position in response regulators enables its sidechain hydroxyl oxygen to hydrogen bond with a phosphoryl group oxygen in the active-site phosphoaspartate [18],[19]. The RapH_A_-induced conformational change in Spo0F_A_ Thr82 is accompanied by the rotation of the Spo0F_A_ His101 sidechain to an internalized position where it enters space vacated by Thr82 (Figure 3A, right panel). It is important to note that while Spo0F is not phosphorylated in the RapH-Spo0F structure, RapH_A_ locks non-phosphorylated Spo0F_A_ in the conformation corresponding to a phosphorylation-stabilized receiver domain, which is characterized by the rotation of both switch residues, as described above, and the movement of the β4-α4 loop towards the active site [20].

Finally, structural alignment of the RapH bound structures of Spo0F_A_ and Spo0F_B_ revealed that they are in different conformations (Figure 3B). The entire β4-α4 loop, all of helix α4, and a portion of the α4-β5 loop are disordered in Spo0F_B_ as evidenced by a lack of interpretable electron density corresponding to these residues. This disorder, particularly in the β4-α4 loop, which, as described below, makes important regulatory contacts with RapH in the RapH_A_-Spo0F_A_ structure, implies that RapH_B_ and Spo0F_B_ may be more loosely associated than RapH_A_ and Spo0F_A_. Consistent with this hypothesis, the Spo0F_B_ switch residues are in the non-phosphorylated conformation. That is, the sidechain of Thr82 is pointing away from the active site, and the His101 side chain is in an external conformation (Figure 3B). Because the Spo0F_B_ β4-α4 loop is disordered and could not be modeled, its conformation could not be directly compared to that of the Spo0F_A_ β4-α4 loop.

### Functional Analysis of the RapH-Spo0F Interface In Vitro

**Figure 5:**
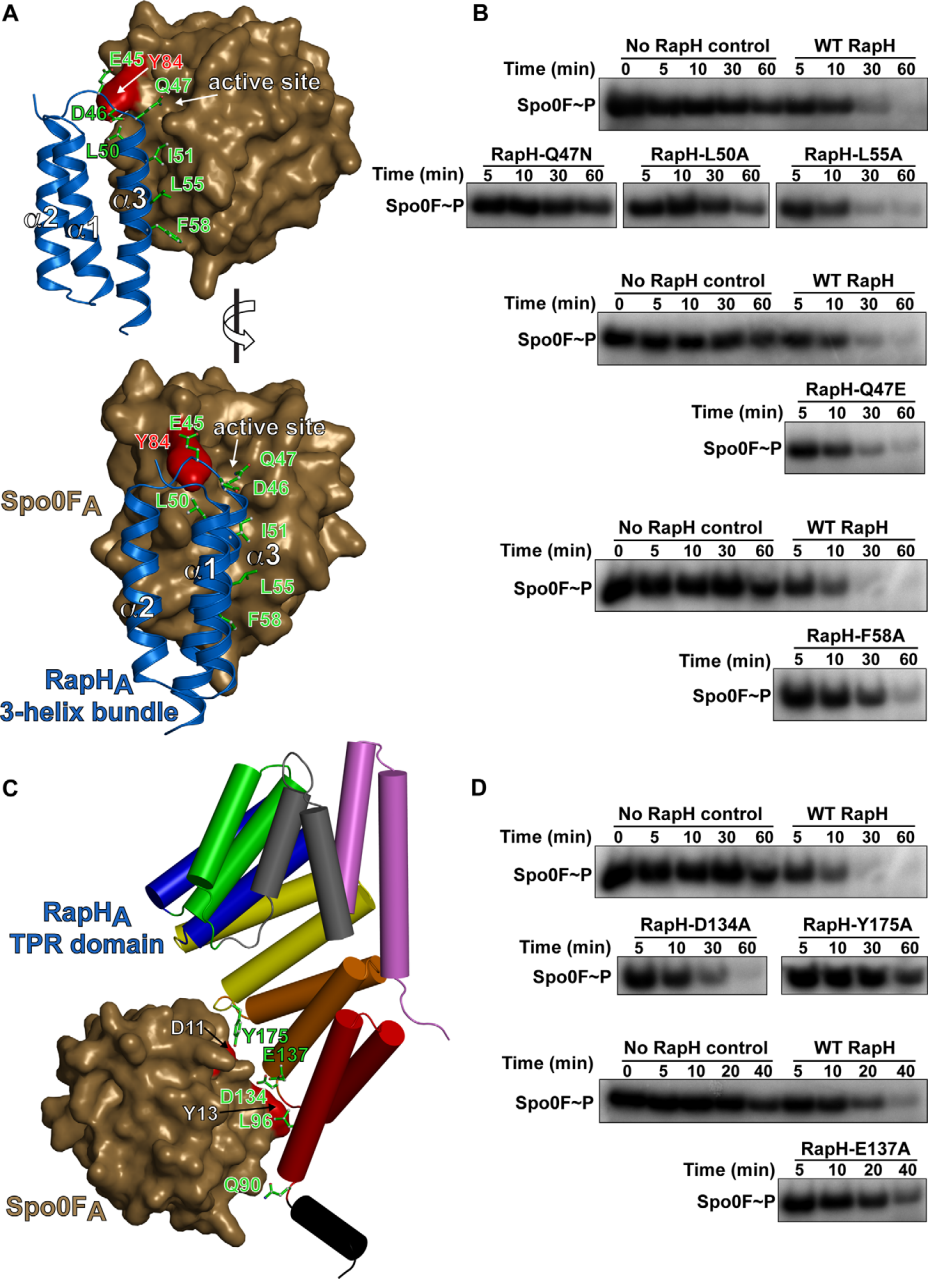
In vitro phosphatase activity of RapH mutants targeting the RapH-Spo0F interface. (A) The RapH_A_ 3-helix bundle (blue) interaction with Spo0F_A_ (brown surface). The phosphatase activity of RapH mutants corresponding to the residues depicted as green sticks was tested. The main-chain nitrogen atom of RapH_A_ residue Asp46 hydrogen bonds with the −OH group of Spo0F_A_ Tyr84. The surface of Spo0F Tyr84 is colored red. (B) The ability of wild-type and mutant RapH mutants to dephosphorylate Spo0F∼P was compared. Above each gel depicting the phosphatase activity of a RapH mutant is a gel containing a wild-type RapH control reaction as well as a no RapH (Spo0F∼P autohydrolysis) control reaction. For the purpose of normalization across gels, identical samples (not shown) from the wild-type RapH control reactions were electrophoresed alongside every RapH mutant reaction. The 5-min time point was ultimately used for across-gel normalization. (C) The RapH_A_ TPR domain (rainbow-colored cylinders) interaction with Spo0F_A_ (brown surface). The phosphatase activity of RapH mutants corresponding to the residues depicted as green sticks was determined. The surfaces of Spo0F Asp11 and Spo0F Tyr13 are colored red. (D) The ability of wild-type and mutant RapH mutants to dephosphorylate Spo0F∼P was compared as in (B). In order to measure the RapH-E137A phosphatase defect, 20 and 40 min time points were evaluated for RapH-E137A in lieu of 30 and 60 time points. Gels are representative of experiments repeated at least three times for each RapH mutant.

To determine the mechanism of RapH phosphatase activity, we systematically explored the functional significance of the RapH-Spo0F interactions observed in the RapH-Spo0F X-ray crystal structure. The phosphatase activity of RapH mutants containing single mutations distributed throughout the RapH-Spo0F interface was first tested in vitro using a biochemical phosphatase assay. Using the in vitro phosphatase assay, we compared the rate of Spo0F dephosphorylation catalyzed by mutant RapH proteins to that of wild-type RapH and no RapH (Spo0F∼P autodephosphorylation) controls (Figure 5).

The RapH N-terminal 3-helix bundle and C-terminal TPR domain interact with Spo0F; therefore, interfacial residues in both of these domains were targeted for mutagenesis (Figure 5A and 5C). The interaction of the RapH_A_ 3-helix bundle domain and Spo0F_A_ buries a total of 968 Å^2^ surface area, and the vast majority of RapH_A_ 3-helix bundle residues buried in the RapH_A_-Spo0F_A_ interface are located in RapH_A_ helix α3 and the α2-α3 loop (Figure 5A). The RapH 3-helix bundle contacts residues in Spo0F_A_ helices α1, α2, and α5, and in the β1-α1, β2-α2, β3-α3, β4-α4, and β5-α5 loops, which surround the Spo0F_A_ active-site pocket (Figures 5A and S3). Initially, we generated RapH proteins containing individual alanine substitutions targeting the following 3-helix bundle residues that we identified as being buried in the Spo0F interface: Glu45, Gln47, Leu50, Leu55, and Phe58 (Figure 5A). Purified RapH-L50A, RapH-L55A, and RapH-F58A were as soluble as wild-type RapH; however, RapH-E45A and RapH-Q47A were largely insoluble. RapH-E45A was not studied further in vitro but was further analyzed in vivo as described below. However, additional effort to obtain highly soluble RapH protein containing an amino acid substitution at Gln47 was warranted because RapH Gln47 inserts into the Spo0F active-site pocket, and we hypothesized that Gln47 plays a particularly important role in RapH function. Therefore, we generated RapH-Q47N and RapH-Q47E which were as soluble as wild-type RapH.

Our biochemical analysis showed that RapH-L50A and RapH-F58A displayed reduced phosphatase activity compared to wild-type RapH (Figure 5B). For example, after 30 min 26% of Spo0F∼P remained in the RapH-L50A reaction, while only 13% remained in the wild-type control (Figure 5B). Similarly, after 30 min 28% of Spo0F∼P remained in the RapH-F58A reaction, while only 3% remained in its wild-type RapH control (Figure 5B). While RapH-L55A displayed a wild-type phenotype, one mutant, RapH-Q47N, had an even greater phosphatase defect than the RapH-L50A and RapH-F58A mutants (Figure 5B). In fact, RapH-Q47N appears to be catalytically dead. That is, the RapH-Q47N reaction is nearly indistinguishable from the no RapH control.

It is important to note that the mutation of RapH Gln47 to asparagine is rather conservative; glutamine and asparagine are uncharged and differ by the presence or absence of a single side-chain carbon atom, respectively. However, shortening the sidechain of RapH Gln47 by the length of one carbon-carbon bond (∼1.5 Å) was sufficient to completely eliminate RapH phosphatase activity in vitro (Figure 5B). Importantly, the RapH-Q47E mutant that replaces glutamine-47 with glutamate displays wild-type phosphatase activity (Figure 5B). As discussed in detail below, we hypothesize that the RapH Gln47 side chain orients a water molecule for nucleophilic attack on the Spo0F aspartylphosphate phosphorous atom. Since RapH-Q47E displays wild-type activity, it appears that an −O^−^ group can substitute functionally for the −NH_2_ group in the Gln47 side chain to coordinate the attacking water.

In addition to interacting with the RapH 3-helix bundle, Spo0F interacts extensively with the RapH TPR domain, burying 968 Å^2^ surface area in RapH_A_-Spo0F_A_ and 953 Å^2^ surface area in RapH_B_-Spo0F_B_ (Figure 5C). While it is possible that Spo0F interactions with the RapH 3-helix bundle are sufficient for RapH phosphatase activity, we hypothesized that the RapH TPR domain contributes important regulatory contacts to the RapH-Spo0F interface as well. To begin to test this hypothesis, we generated RapH-D134A, RapH-E137A, and RapH-Y175A and measured the phosphatase activity of these mutants in vitro (Figure 5D). RapH-D134A, RapH-E137A, and RapH-Y175A were significantly impaired for phosphatase function (Figure 5D). After 30 min 13% and 54% of Spo0F∼P remained in the RapH-D134A and RapH-Y175A reactions, respectively, while only 3% remained in their wild-type control (Figure 5D). Similarly, after 40 min 15% of Spo0F∼P remained in the RapH-E137A reaction while only 7% remained in its wild-type control. Therefore, we conclude that Spo0F interactions with both the RapH N-terminal 3-helix bundle and the C-terminal TPR domain are required for wild-type phosphatase activity in vitro.

### Functional Analysis of the RapH-Spo0F Interface In Vivo

**Figure 6:**
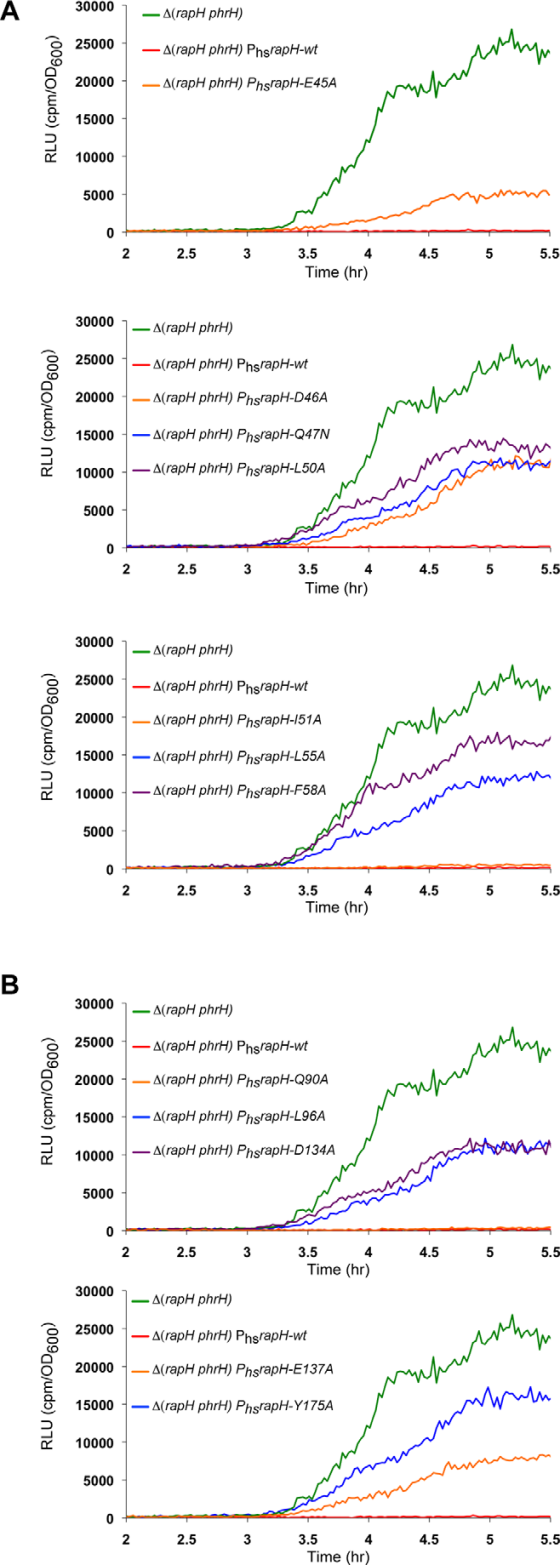
In vivo activity of RapH mutants targeting the RapH-Spo0F interface. RapH activity was measured as a function of P*spoIIG*::luc. (A) In vivo analysis of RapH proteins containing mutations in the 3-helix bundle lying in the Spo0F interface as depicted in Figure 5A. (B) In vivo analysis of RapH proteins containing mutations in the TPR domain lying in the Spo0F interface as depicted in Figure 5C. Each curve is representative of at least three independent experiments performed in duplicate. (A and B) Wild-type and mutant *rapH* overexpression was controlled by the IPTG-inducible promoter P_hyperspank_. RLU, Relative Luminescence Units.

To confirm the physiological relevance of our X-ray crystallographic and biochemical results, we measured wild-type and mutant RapH protein activity in *B. subtilis* as a function of Spo0A activation using a luciferase reporter gene under the control of the Spo0A-driven promoter P*spoIIG* (Figure 6). Since RapH dephosphorylates Spo0F, resulting in the repression of Spo0A transcriptional activity, overexpression of wild-type RapH inhibits the expression of the P*spoIIG*-driven luciferase reporter (Figure 6A and 6B). Conversely, less active RapH mutants will display elevated levels of luciferase expression from the P*spoIIG* reporter.

We first tested the effects of the RapH mutants described above on Spo0F dephosphorylation in *B. subtilis.* RapH-L55A, which displays wild-type activity in vitro, showed reduced activity in vivo; however, this appears to be the result of its significantly reduced expression compared to wild-type RapH (Figures 6A and S4). In close agreement with our biochemical studies, RapH-Q47N, RapH-L50A, and RapH-F58A displayed reduced activity in vivo (Figure 6A). Western blotting confirmed that all of these RapH mutants were produced at levels comparable to wild-type RapH in *B. subtilis* (Figure S4).

To further extend our analysis of the RapH-Spo0F interaction surface and to identify additional regulatory contacts, we examined a number of additional RapH proteins containing 3-helix bundle mutations lying in the Spo0F interface (Figures 5A, 6A, and S4). While RapH-I51A displayed wild-type RapH function, RapH-E45A and RapH-D46A displayed drastically reduced activity in vivo (Figure 6A). Thus, the RapH_A_-Spo0F_A_ complex shows that RapH residues Glu45, Asp46, and Leu50 surround the side chain of Spo0F Tyr84, and our functional analysis shows that mutating any of these RapH residues to alanine inhibits RapH activity (Figures 5A, 5B, and 6A). It is also important to note that the identity of the Spo0F_A_ Tyr84 binding pocket residues is highly conserved in all Rap proteins known to dephosphorylate Spo0F and that both RapH Glu45 and the Spo0F Tyr84-containing β4-α4 loop are disordered in RapH_B_-Spo0F_B_ (Figures 3B and 4). Thus, the observed interaction of RapH with Tyr84 appears to be important for locking Spo0F in the conformation observed in RapH_A_-Spo0F_A_ and required for wild-type levels of RapH activity in vivo and in vitro.

In agreement with our biochemical results, RapH proteins containing mutations in residues lining the surface of the RapH TPR domain-Spo0F interface—L96A, D134A, E137A, and Y175A—displayed reduced activity in *B. subtilis* (Figures 5C, 6B, and S4). These results were not surprising because RapH residue Leu96, like Asp134 and Glu137, contributes to the formation of the Spo0F Tyr13 binding pocket, and Spo0F Tyr13 was previously shown to be important for Rap protein-mediated dephosphorylation of Spo0F (Figure 5C; [16]). Furthermore, the RapH-Spo0F crystal structure shows that RapH residue Tyr175 hydrogen bonds to the Spo0F Mg^2+^-coordinating residue Asp11 and may help stabilize Mg^2+^ coordination, which is required for dephosphorylation (Figure 5C; [15]). To complete our mutagenic analysis of the RapH-Spo0F interface, we also tested RapH-Q90A, which displayed wild-type activity in vivo (Figure 6B). RapH Gln90 is located in the loop connecting helix α4 to TPR1 and lies on the periphery of the RapH-Spo0F interface (Figure 5C). Including this mutation in our in vivo analysis resulted in complete mutational coverage of the RapH-Spo0F interface. Ultimately, we tested the effects of at least one mutation located in every RapH helix and loop in the Spo0F interface. The results of both the in vivo and in vitro functional analysis are summarized in Figure 4.

### RapH Obstructs Phosphotransfer to and from Spo0F

Taken together, our X-ray crystallographic, biochemical, and genetic results suggest that the RapH-Spo0F interfaces we identified in the RapH-Spo0F crystal structure are physiologically important. However, we found it curious that RapH mutants displaying a severe loss-of-function phenotype in vitro, such as RapH-Q47N, which is catalytically dead, still inhibited, at least to some degree, the flow of phosphoryl groups along the sporulation phosphorelay in vivo (Figures 5B and 6A). Interestingly, the RapH-Spo0F structure revealed that RapH binds to a Spo0F surface containing numerous residues previously shown to be important for its interaction with KinA and Spo0B (Figure S2; [21]). Therefore, we hypothesized that catalytically dead RapH mutants, and potentially wild-type RapH as well, could bind to Spo0F and obstruct its phosphorylation by the sporulation histidine kinases and phosphotransfer to Spo0B.

**Figure 7:**
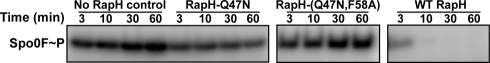
Rap proteins inhibit phosphotransfer from KinA to Spo0F. RapH-Q47N does not catalyze Spo0F dephosphorylation (see Figure 5B), but it sterically inhibits phosphotransfer from KinA to Spo0F. RapH-Q47N,F58A contains the catalysis-eradicating Q47N active-site mutation as well as the RapH-Spo0F interfacial mutation, F58A. RapH-Q47N,F58A does not dephosphorylate Spo0F or inhibit phosphotransfer from KinA to Spo0F. For the purpose of normalization across gels, identical samples (not shown) from the no RapH control reaction were electrophoresed alongside every RapH-containing reaction. The 60-min time point was ultimately used for across-gel normalization. The gels are representative of the results from three independent phosphotransfer experiments.

To test this hypothesis, we mixed Spo0F with the catalytically dead RapH-Q47N mutant and found that it inhibited Spo0F phosphorylation by KinA in a phosphotransfer assay when KinA, RapH-Q47N, and Spo0F were present in a molar ratio of 0.006∶0.4∶1 (Figure 7). When the KinA:RapH-Q47N:Spo0F molar ratio was 0.006∶4∶1, phosphotransfer from KinA to Spo0F was nearly undetectable even after 1 h (unpublished data). Not surprisingly, RapH-Q47N also blocked phosphotransfer from Spo0F to Spo0B in vitro (unpublished data). Furthermore, when we more drastically disrupted the RapH-Spo0F interaction by incorporating another RapH-Spo0F interface mutation, RapH-F58A, into the RapH-Q47N mutant, we found that the double mutation relaxed the RapH-mediated inhibition of phosphotransfer from KinA to Spo0F and from Spo0F to Spo0B (Figure 7 and unpublished data). Thus, RapH-Q47N can bind Spo0F and restrict KinA and Spo0B access to the Spo0F active site. Finally, while RapH residue Gln47 is absolutely required for RapH to dephosphorylate Spo0F, our in vivo and in vitro results suggest that this residue might not contribute significantly to the RapH-Spo0F binding energy.

### Sequence-Based Assignment of Rap Phosphatase Activity

In the absence of a Rap protein-Spo0F X-ray crystal structure, sequence-based prediction of Rap protein function was not previously possible. Mapping the sequence conservation of *B. subtilis* RapA, RapB, RapE, and RapH, as well as *B. anthracis* Rap proteins BXA0205 and BA3790, which have been shown to dephosphorylate *B. subtilis* Spo0F, onto the structure of RapH revealed 18 highly conserved positions in the RapH-Spo0F interface (Figure 4; [22]). We hypothesized that these residues occupy functionally important positions and that we could predict whether other Rap proteins are Spo0F phosphatases based on their amino acid sequence identity in these positions.

To test this hypothesis, we analyzed the sequence of other *Bacillus* Rap proteins and determined that *B. subtilis* RapJ, among other Rap proteins, contains a high degree of sequence similarity in these interfacial residues (Figure 4). In fact, RapJ encodes a residue that is identical to a residue in one of the other known Spo0F Rap phosphatases in 15 of the 18 highly conserved interfacial positions. The least well-conserved RapJ residues in the 18 highly conserved interfacial positions are Leu40, Asn46, and Phe53, corresponding to RapH residues Ile40, Asp46, and Tyr53, respectively (Figure 4). Modeling leucine in place of Ile40 in RapH showed that leucine could substitute for its positional isomer isoleucine without introducing atomic clashes (unpublished data). Furthermore, the RapH-Spo0F structure showed that RapH Asp46 makes only main-chain contacts with Spo0F, so it seemed likely that a Rap phosphatase could tolerate asparagine in this position. Finally, the hydroxyl moiety of RapH Tyr53 is not involved in a hydrogen bond with Spo0F and its aromatic ring appears to be more important to the Spo0F interaction; thus Phe53 in RapJ would be unlikely to disrupt phosphatase function.

In contrast with RapJ, the non-phosphatase Rap protein RapD conserves only 6 of the 18 highly conserved interfacial positions. Modeling RapD residues in place of the conserved RapH interfacial residues showed that many of them would clash with Spo0F (unpublished data). For example, RapD contains tryptophan at residue 50 where RapH contains leucine. RapD also encodes alanine at residue 58 where RapH contains phenylalanine, and alanine substituted for phenylalanine at this position in RapH resulted in a loss of function in vitro and in vivo. Furthermore, RapD contains threonine at the position equivalent to the RapH catalytic residue Gln47.

**Figure 8:**
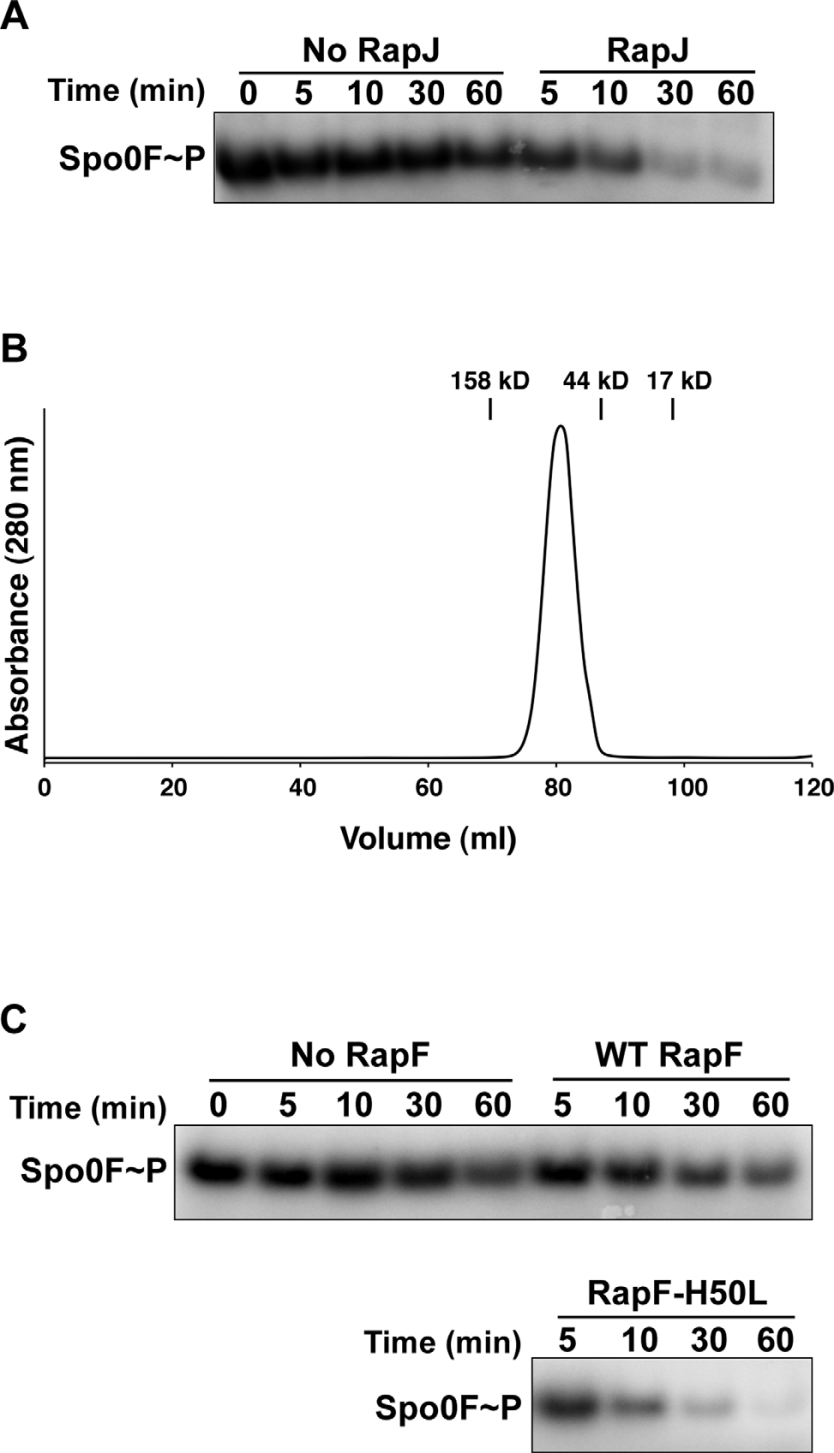
Predicting and engineering Rap phosphatase activity. (A) RapJ dephosphorylates Spo0F∼P. The gel is representative of phosphatase assays repeated four times. (B) Size exclusion chromatography shows that RapJ (MW_theor_ 44.4 kDa) forms RapJ_2_ dimers (MW_exper_ 92.0 kDa) in solution. The peak positions of gel filtration standards are indicated by vertical lines above the absorbance traces. (C) RapF-H50L dephosphorylates Spo0F∼P while wild-type RapF does not. The gels are representative of phosphatase assays repeated three times.

To test our prediction that RapJ is a Spo0F phosphatase, we overexpressed and purified RapJ and evaluated its ability to dephosphorylate Spo0F in vitro. Indeed, RapJ dephosphorylated Spo0F (Figure 8A). After 30 min only 4% of Spo0F∼P remained in the RapJ reaction, while 60% remained in the no RapJ control (Figure 8A). This result is consistent with microarray data showing that RapJ overproduction in *B. subtilis* affects the expression of 20 Spo0A-regulated operons [23]. Finally, like every other Rap protein that we have tested to date, RapJ is dimeric as determined by gel filtration chromatography (Figure 8B). Thus, insight obtained from the RapH-Spo0F X-ray crystal structure, together with biochemical and genetic analysis of the observed RapH-Spo0F interactions, has enabled us to assign Rap phosphatase function based on sequence alone.

### Engineering De Novo Rap Phosphatase Activity

To rigorously test our understanding of the structural requirements for Rap phosphatase activity and also to confirm that we have identified the biologically important Rap-Spo0F interface, we engineered a non-phosphatase Rap protein to dephosphorylate Spo0F. Sequence alignment revealed that the non-phosphatase protein RapF, which targets the DNA binding domain of ComA and does not dephosphorylate its REC domain [24],[25], contains a glutamine at residue 47 which aligns with the RapH catalytic residue Gln47. Additionally, RapF contains a residue identical to at least one of the other Spo0F Rap phosphatases in all of the highly conserved interfacial residues with only two exceptions (Table S2). More specifically, RapF contains histidine and lysine where RapH contains Leu50 and Ser133, respectively.

We predicted that lysine could be tolerated in RapF at the position corresponding to RapH Ser133 without disrupting potential Spo0F phosphatase activity because (1) the RapH_A_ Ser133 side chain −OH points away from the Spo0F_A_ interface and only the Cβ side-chain atom and main-chain atoms are buried in the Spo0F_A_ interface, (2) in RapH_B_ only main-chain atoms belonging to Ser133 are buried in the Spo0F_B_ interface, and (3) modeling lysine at RapH residue 133 revealed that nearly every lysine side-chain rotamer could be tolerated without clashing with Spo0F. However, modeling histidine at RapH Leu50 in the RapH-Spo0F structure showed that every histidine side-chain rotamer clashed with Spo0F or RapH itself (unpublished data). Based on these findings, we hypothesized that this one residue, His50, disables Spo0F phosphatase function in RapF.

To test the above hypothesis, we purified RapF-H50L and measured its phosphatase activity in vitro. Indeed, RapF-H50L dephosphorylates Spo0F, while wild-type RapF does not. After 30 min only 16% of Spo0F∼P remained in the RapF-H50L reaction while 54% remained in the wild-type RapF control (Figure 8C). These results, along with our structural analysis, reveal the mechanistic basis underlying the inability of wild-type RapF to dephosphorylate Spo0F and confirm that we have identified the biologically important Rap protein surface and many of the structural determinants required for Rap phosphatase activity. Furthermore, this represents an interesting and rare example of rational surface design that generates new target protein specificity and de novo enzymatic function.

## Discussion

### Mechanism of Rap Activity

Our X-ray crystallographic, biochemical, and genetic studies suggest that RapH_A_-Spo0F_A_ represents a complex formed in *B. subtilis* when RapH binds to phosphorylated Spo0F. Conversely, RapH_B_-Spo0F_B_ depicts a conformation adopted immediately following RapH-mediated Spo0F dephosphorylation. The disorder we observed in residues comprising the RapH_B_-Spo0F_B_ interface suggests that the RapH_B_-Spo0F_B_ structure represents a destabilized RapH-Spo0F complex. Destabilization of the RapH-Spo0F interaction following Spo0F dephosphorylation could enable RapH to dissociate from non-phosphorylated Spo0F, freeing RapH to participate in another round of Spo0F dephosphorylation.

RapH and a catalytically dead RapH mutant that cannot dephosphorylate Spo0F inhibited phosphotransfer from KinA to Spo0B (Figure 7 and unpublished data). Because receiver domains readily adopt the phosphorylated conformation even in the absence of phosphorylation [26],[27],[28], we hypothesize that RapH could be binding to either phosphorylated or non-phosphorylated Spo0F and inhibiting the sporulation phosphorelay by sterically limiting kinase and phosphotransferase access to the Spo0F active site. Consistent with this hypothesis, RapA was previously shown to interact with phosphorylated and non-phosphorylated Spo0F, albeit the interaction with phosphorylated Spo0F appeared to be more stable than the interaction with non-phosphorylated Spo0F [29]. We expect that steric inhibition of Spo0F phosphorylation would have the most significant biological effect when the concentration of Spo0F and KinA are limiting and Rap proteins are in excess. In *B. subtilis* alone, RapA, RapB, RapE, RapH, and RapJ all dephosphorylate Spo0F and presumably block access to the Spo0F active site. Therefore, it seems possible that the steric effects that Rap proteins have on Spo0F phosphotransfer in addition to their phosphatase activity may be physiologically relevant. Consistent with this hypothesis, overexpressed catalytically dead RapH inhibited phosphotransfer in *B. subtilis* cells (Figure 6A).

**Figure 9:**
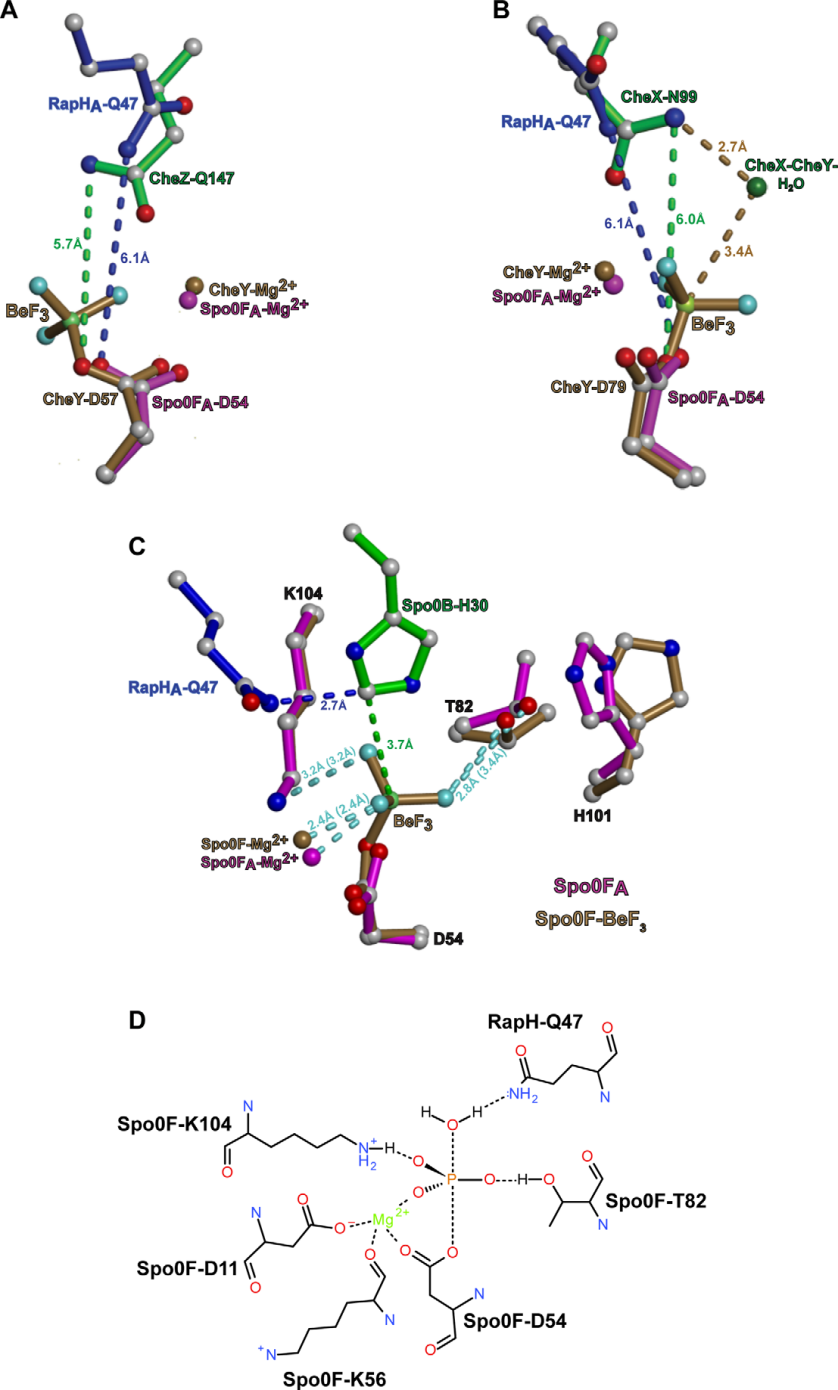
Structural comparison of response regulator phosphatase and phosphotransferase active sites, and a model of RapH-Spo0F dephosphorylation chemistry. The Spo0F_A_ molecule of RapH_A_-Spo0F_A_ was aligned with the CheY molecule of CheZ-BeF_3_-CheY (PDB ID 1KMI) (panel A), the CheY molecule of CheX-BeF_3_-CheY (PDB ID 3HZH) (panel B), and the Spo0F molecule of Spo0B-BeF_3_-Spo0F (PDB ID 2FTK) (panel C). Structural alignments were performed using DaliLite [45] and PyMol [41]. (Panel A) The amide nitrogens of RapH_A_ Q47 and CheZ Q147 are similarly positioned relative to their target receiver domain active-site aspartates. The RapH_A_ Q47 and CheZ Q147 amide nitrogens are 5.9 Å and 5.7 Å, respectively, from the sites of phosphoryl group addition in their target receiver domains. (Panel B) The amide nitrogens of RapH_A_ Q47 and CheX N99 are similarly positioned relative to their target receiver domain active-site aspartates. The RapH_A_ Q47 and CheX N99 amide nitrogens are 5.9 Å and 6.0 Å, respectively, from the sites of phosphoryl group addition in their target receiver domains. The water molecule that attacks the CheY phosphoaspartate phosphorous atom in the CheX-BeF_3_-CheY structure is shown and reveals approximately where the attacking water may be positioned during RapH mediated dephosphorylation of Spo0F. (C) The phosphoryl group mimic beryllium fluoride in the Spo0B-BeF_3_-Spo0F structure shows where the phosphoryl group would lie in a RapH-Spo0F∼P complex. The distances between the BeF_3_ fluorine atoms in the Spo0B-BeF_3_-Spo0F structure and the atoms that are positioned to coordinate them in the structures of Spo0F-BeF_3_-Spo0B and RapH_A_-Spo0F_A_ are depicted as light blue dashed lines. The distances between the fluorine atoms and the coordinating Spo0F_A_ atoms are in parentheses. The Spo0F_A_ His101 side chain is in an internal position in the RapH_A_ complex, while the Spo0F His101 side chain in the Spo0B-BeF_3_ complex is not. This is consistent with the active-site proximal and active-site distal conformations of their Spo0F β4-α4 loops (not shown), respectively. (D) A trigonal bipyramidal phosphoryl group and attacking water were modeled into the RapH-Spo0F active site to generate a graphical depiction of the dephosphorylation transition state. Amino acid positions reflect their conformations in RapH_A_-Spo0F_A_. Bonds are depicted as black dashed lines.

The RapH-Spo0F structure revealed that the sidechain of RapH Gln47 inserts into the Spo0F active site (Figure 5A). Importantly, without exception, glutamine is conserved at this position in Rap proteins known to dephosphorylate Spo0F (Figure 4), and replacing RapH Gln47 with asparagine was sufficient to completely eliminate phosphatase activity (Figures 5B). We propose that RapH Gln47 orients water for direct in-line hydrolytic attack on the Spo0F phosphoaspartate-54 phosphorous atom (Figure 9D).

Consistent with our proposed model of RapH function and the critical role that RapH Gln47 plays in dephosphorylating the response regulator Spo0F, the CheZ and CheC/CheX/FliY families of response regulator phosphatases insert a conserved glutamine and asparagine, respectively, into their target response regulator active sites where they orient water for nucleophilic attack on the phosphoaspartate (reviewed in [7]). Aligning the Spo0F molecule of RapH_A_-Spo0F_A_ with the CheY molecules of the CheZ-BeF_3_-CheY and CheX-BeF_3_-CheY structures shows that the amide nitrogens in RapH_A_ Gln47, CheZ Gln147, and CheX Asn99 are positioned at nearly identical distances—6.1 Å, 5.7 Å, and 6.0 Å, respectively—from their corresponding receiver domain active-site aspartate (Figure 9A and 9B). Consistent with the hypothesis that RapH_B_-Spo0F_B_ represents a conformation adopted following RapH-mediated Spo0F dephosphorylation, the amide nitrogen in RapH_B_ Gln47 is located 1.1 Å farther away from the Spo0F_B_ active-site aspartate than the RapH_A_ Gln47 amide nitrogen is from the Spo0F_A_ active-site aspartate.

Furthermore, aligning the Spo0F molecules of the RapH_A_-Spo0F_A_ and Spo0B-BeF_3_-Spo0F structures illustrates approximately where RapH Gln47 would position water for nucleophilic attack on the phosphoaspartate phosphorous atom (Figure 9C; [19]). More specifically, a water molecule hydrogen bonded to RapH Gln47 Nε2 and lying in a position near those occupied by Spo0B His30 Cε1 and Nε2 would be perfectly positioned for in-line attack on the Spo0F phosphoaspartate phosphorous atom (Figure 9C). We suspect that direct observation of the attacking water molecule in the crystal structure will require crystallizing the RapH-Spo0F-Mg^2+^ complex in the presence of a non-hydrolyzable phosphoryl mimic such as beryllium fluoride or vanadate.

Consistent with our proposed mechanism of RapH-mediated Spo0F dephosphorylation, there are no data suggesting that Spo0F transfers phosphoryl groups to RapH resulting in the formation of a phosphoenzyme intermediate. We detect no RapH phosphoenzyme intermediate in our in vitro assays, and it was previously shown that RapB dephosphorylation of Spo0F was not inhibited by the protein tyrosine phosphatase transition-state inhibitor vanadate ([16]; and unpublished data).

While the catalytic mechanism of RapH-mediated Spo0F dephosphorylation appears to be similar to the one employed by CheZ and CheC/CheX/FliY family members, its overall interaction with its target response regulator is different. Along with the catalytic amide-containing sidechain, the CheZ and CheC/CheX/FliY phosphatases insert an acidic amino acid sidechain into the receiver domain active site where it forms a salt bridge with the active site lysine equivalent to Spo0F Lys104 [30],[31],[32]. Insertion of the acidic residue into the receiver domain active site is required for wild-type CheZ and CheC/CheX/FliY family phosphatase activity. RapH inserts only an amide-containing side chain into the target response regulator active site, and the RapH-Spo0F structure shows that a salt bridge between RapH and Spo0F Lys104 is not required for RapH phosphatase activity.

Finally, while the explicit mechanistic basis of Spo0E family phosphatase function is unknown, genetic and biochemical data suggest that an acidic residue may be coordinating the water for hydrolysis rather than an amide-containing side chain [33]. A RapH mutant containing glutamate in place of the catalytic residue, Gln47, could dephosphorylate Spo0F (Figure 5B). Together, the structural and functional data presented here suggest that glutamine or glutamate at the position equivalent to RapH Gln47 is a requirement for Rap protein-mediated Spo0F dephosphorylation.

### The RapH-Spo0F Structure Supports T-loop-Y Coupling

In the T-loop-Y model of receiver domain allostery, the conformation of the hydroxyl-containing switch residue is coupled to the conformation of the β4-α4 loop, and the β4-α4 loop gates conformational changes in the aromatic switch residue in β5 [34]. More specifically, when the β4-α4 loop is in an active site proximal conformation, favored when the hydroxyl switch residue rotates toward the active site, e.g. in response to phosphorylation, it causes the internalization of the aromatic switch residue. Internalization of this aromatic residue regulates intra- and inter-molecular protein-protein interactions that occur at the α4-β5-α5 surface of receiver domains (reviewed in [35]). Interestingly, the existing data support a T-loop-Y coupling model that is directional [34]. That is, a β4-α4 loop oriented toward the active site dictates the internalization of the aromatic switch residue side chain; however, internalization of the aromatic switch residue does not restrict the conformation of the β4-α4 loop.

Aligning Spo0F_A_ with the structure of Spo0F bound to BeF_3_ and Spo0B showed that the Thr82 switch residues are in similar positions pointing toward the active site in both models (Figure 9C). However, His101 is in an internal position in the RapH_A_-Spo0F_A_ structure, while it is in an external position in the Spo0B-BeF_3_-Spo0F structure (Figure 9C). In support of the T-loop-Y coupling model, it appears that the internalization of His101 in RapH_A_-Spo0F_A_, likely resulting from RapH-mediated stabilization of the β4-α4 loop in the active-site proximal conformation, is not observed in the Spo0F-BeF_3_-Spo0B structure. Also consistent with this model, the Spo0F_B_ His101 side chain is free to adopt an external conformation, probably as a result of the disordered Spo0F_B_ β4-α4 loop.

## Conclusion

The X-ray crystal structure of the response regulator phosphatase RapH in complex with one of its cellular targets, Spo0F, combined with extensive biochemical and genetic analysis of RapH mutants, reveals the mechanism of Rap-mediated phosphatase activity along with many of the structural requirements for Rap phosphatase function. The approach presented here that enabled us to assign Spo0F phosphatase function to RapJ based only on its amino acid sequence could be used to predict the target specificity of non-Spo0F phosphatase Rap proteins following the determination of structures of representative complexes of the Rap proteins bound to their targets. Furthermore, identifying the non-Spo0F phosphatase Rap protein residues important for target recognition will facilitate (1) the identification of Rap proteins whose sequences suggest that they may not recognize previously identified substrates, (2) the identification of new Rap protein targets, and (3) rational engineering of de novo Rap protein target specificity. The process of developing Rap proteins with engineered target specificity will not only provide valuable insight into the principles governing protein-protein interaction but will also provide valuable reagents for studying bacterial signal transduction. Finally, insight gleaned from our structure-function analysis of RapH-Spo0F, along with additional studies of other Rap proteins in complex with their response regulators, could be used to develop new classes of drugs that disrupt the flow of information along bacterial phosphotransfer signaling pathways by mimicking the antagonistic effects of Rap proteins on response regulators.

## Materials and Methods

### Protein Production for X-Ray Crystallography

*RapH* was PCR-amplified from *B. subtilis* strain 168 chromosomal DNA using Phusion High-Fidelity DNA Polymerase (NEB, USA) and the oligonucleotide pair RapH-BamHI and RapH-NotI (Table S3). The PCR product was cloned in the BamHI and NotI restriction sites of pGEX4T1 (GE Healthcare) to yield pGEXH1. GST-RapH was overexpressed in *E. coli* strain BL21 by first growing the cells at 37°C in LB medium containing 100 µM ampicillin to OD_600_ = 0.6 and then inducing expression with 0.1 mM isopropyl β-D thiogalactopyranoside (IPTG) for 16 h at 16°C. All subsequent purification steps were carried out at 4°C. The cells were collected by centrifugation and lysed in buffer A (20 mM Tris-HCl (pH 8.0), 250 mM NaCl, 50 mM KCl, 10 mM MgCl_2_, 5 mM DTT, 10% glycerol) supplemented with 1 µM Pepstatin, 1 µM Leupeptin, 20 µg/ml DNase, and 1 mM phenylmethanesulfonyl fluoride (PMSF). The lysate supernatant was applied to Glutathione Uniflow Resin (Clontech) equilibrated in buffer A. The resin was then washed and resuspended in buffer A, and thrombin was added at 0.3 mg/ml glutathione bed volume. Overnight incubation at 4°C resulted in complete cleavage of RapH from the GST affinity tag as determined by SDS-PAGE. Following thrombin digestion, RapH (residues 1–376) contained two heterologous N-terminal residues (Gly-Ser) derived from the thrombin cleavage signal. RapH was eluted with buffer A and diluted 3-fold with buffer B (20 mM Tris-HCl (pH 8.0), 10 mM MgCl_2_, 5 mM DTT, and 10% glycerol), passed through a 0.45 µm filter, and loaded onto an anion exchange column (Source 15Q; GE Healthcare) equilibrated in buffer B containing 50 mM KCl. RapH was then eluted in a 50–1,000 mM KCl gradient of buffer B. Fractions containing RapH were pooled, concentrated by ultrafiltration through a 30 kDa filter, and further purified by gel filtration using a Superdex 200 (GE Healthcare) 16/70 column equilibrated in buffer C (20 mM Tris-HCl (pH 8.0), 150 mM KCl, 5 mM MgCl_2_, and 5 mM DTT). RapH was concentrated to 14 mg/ml and stored at −80°C.

*Spo0F* was PCR-amplified from *B. subtilis* strain 168 chromosomal DNA using Phusion High-Fidelity DNA Polymerase and the oligonucleotide pair Spo0F-BamHI and Spo0F-NotI (Table S3). The PCR product was cloned in the BamHI and NotI restriction sites of pETDuet-1 (GE Healthcare) to yield pETDuetF1. The Spo0F-D54E expression vector was obtained by site-directed mutagenesis of pETDuetF1 using primers D54E_Spo0F_T and D54E_Spo0F_B to give pETD54E (Table S3). Spo0F and Spo0F-D54E were expressed in *E. coli* strain BL21(DE3) by first growing the cells at 37°C in LB medium containing 100 µM ampicillin to OD_600_ = 0.5 and then inducing expression with 1 mM IPTG for 16 h at 25°C. All subsequent purification steps were carried out at 4°C. Cells were collected by centrifugation and lysed in buffer D (25 mM Tris-HCl (pH 8.0), 10 mM KCl, 40 mM MgCl_2_, and 5 mM β-mercaptoethanol (β-ME)) supplemented with 1 µM Pepstatin, 1 µM Leupeptin, 20 µg/ml DNase, and 1 mM PMSF. The supernatant was applied to Glutathione Uniflow Resin equilibrated in buffer D. Following a wash with three bed volumes of buffer D, the resin was incubated with thrombin at 0.3 mg/ml glutathione resin and incubated overnight at 4°C. SDS-PAGE analysis confirmed that the GST tag was removed, and Spo0F was eluted with buffer D. Following thrombin digestion, Spo0F (residues 1–124) contained two heterologous N-terminal residues (Gly-Ser) derived from the thrombin cleavage signal. The protein was then further purified using anion exchange (Source 15Q) and HiTrap Heparin HP (GE Healthcare) columns arranged in series and equilibrated in buffer D. Spo0F eluted in the flow-through was concentrated by ultrafiltration using a 10 kDa cutoff filter, and subjected to gel filtration using a Superdex 200 16/70 column equilibrated with buffer E (20 mM Tris-HCl (pH 8.0), 50 mM KCl, and 5 mM MgCl_2_). Selenomethionyl Spo0F-D54E was purified in an identical manner with the exception that buffer D contained 120 mM MgCl_2_. Spo0F and selenomethionyl Spo0F-D54E eluted exclusively as monomers were concentrated to 7.5 mg/ml and stored at −80°C.

### Crystallization and Diffraction Data Collection

RapH-Spo0F crystals were produced by the vapor diffusion method at 20°C using a 1∶1 mixture of protein (7.5 mg/ml RapH and 3.5 mg/ml Spo0F in 20 mM Tris-HCl (pH 8.0), 100 mM KCl, 2.5 mM DTT, 5 mM MgCl_2_) and well solution (20% [w/v] PEG 3350, 200 mM lithium sulphate, and 100 mM Bis-Tris (pH 6.0)). RapH-Spo0F crystals were cryoprotected in mother liquor solution containing 16.66 mM potassium phosphate buffer (pH 6.0), 17.2 mM MgCl_2_, and 12.5% glycerol for 20 min. Crystals containing RapH and selenomethionyl Spo0F(D54E) were grown under similar conditions to the native RapH-Spo0F crystals with the exception that the Bis-Tris was at pH 5.8. Crystals containing the selenomethionyl derivatized Spo0F protein were cryoprotected for 2–3 s in their mother liquor solutions containing 10% glycerol, 3.5 mM MgCl_2_, and 1 mM DTT. Single-wavelength anomolous dispersion (SAD) and native data on nitrogen-cooled crystals were collected at NSLS beamline X29 and processed using the HKL software package [36].

### Structure Determination and Refinement

The RapH-Spo0F crystal structure was determined by the SAD method using crystals of native RapH bound to selenomethionyl Spo0F(D54E) that are isomorphous to native RapH-Spo0F crystals. PHENIX (AutoSol) was used to locate heavy atom positions, calculate phases, and generate an initial model at 2.70 Å resolution [37]. This model was then refined against 2.20 Å native data in PHENIX. The final model was generated through iterative cycles of building in COOT [38] and refinement in PHENIX. The RapH and Spo0F models were built de novo into the SAD-phased map. The earliest rounds of refinement in PHENIX employed simulated annealing as well as rigid body, individual atomic coordinate, and individual B-factor refinement. The later rounds of refinement in PHENIX employed individual atomic coordinate and individual B-factor refinement, as well as a TLS model whose initial parameters were guided by the TLS Motion Determination (TLSMD) server [39]. During the final rounds of refinement in PHENIX, the stereochemistry and ADP weights were optimized, i.e. the weights yielding the lowest R_free_ value were used for refinement. Water molecules were added only after the RapH and Spo0F models were complete. The vast majority of the modeled water molecules are bound to RapH_A_ or RapH_B_. The water molecule B-factors are comparable to those of the residues they are bound to. Insufficient electron density was observed for the following residues and they were omitted from the model: RapH_A_ 1–3 and 69–76; RapH_B_ 1–3, 27–45, 67–85, 292–294; Spo0F_A_ 1–3 and 120–124; and Spo0F_B_ 1–3, 83–98, and 120–124. Two molecules each of glycerol, sulfate, and Mg^2+^ were built into clear electron density during the final stages of refinement. Ramachandran statistics were calculated in Molprobity [40]. Molecular graphics were produced with PyMOL [41].

### Protein Production for Phosphatase and Phosphotransfer Assays

RapH and RapF mutant expression vectors were generated by site-directed mutagenesis of pGEXH1 and pGEXRapF using the ChangeIT Mutagenesis Kit (USB) (Table S3). The expression vectors contained only the desired mutations, as determined by DNA sequencing. RapH, RapH mutants, RapF, and RapF-H50L were overexpressed and purified as described for the RapH protein used for crystallography with the exception that dialysis was performed rather than gel filtration to exchange the SourceQ column buffers for buffer C containing 10% glycerol.

*rapJ* was amplified from *B. subtilis* strain 168 genomic DNA using Phusion High-Fidelity DNA Polymerase and the primer pair RapJ-Fwd and RapJ-Rev (Table S3). The PCR product was cloned into the SapI and XhoI sites of pTB146 using the In-Fusion method (Clontech) to give pTB146J [42]. His-Sumo-RapJ was overexpressed and purified using the RapH purification protocol described above with the exceptions that buffer A had 10 mM β-ME instead of 5 mM DTT, and instead of being applied to glutathione resin the lysate supernatant was applied to Ni-NTA agarose (Qiagen) equilibrated in buffer A. The Ni-NTA resin was then washed in buffer A and resuspended in 65 mM Tris-HCl (pH 8.0), 325 mM NaCl, 35 mM KCl, 7 mM MgCl_2_, 3.5 mM DTT, 10% glycerol, and 0.2% NP-40. Sumo protease was then added at 4 mg/ml Ni-NTA resin and incubated at 25°C for 2 h. RapJ contained no heterologous residues following removal of the N-terminal His-Sumo fusion. RapJ was then eluted, purified by gel filtration (Superdex 200), and concentrated to 75 mg/ml. Aliquots were stored at –80°C.

*Spo0F* was amplified from pGEXF using primers Spo0F-NdeI and Spo0F-XhoI, digested with NdeI and XhoI, and cloned into the pET21b (Novagen) NdeI and XhoI sites to give pET21bF (Table S3). Spo0F containing a C-terminal fusion to hexahistidine was then purified as previously described [21].

*KinA* was amplified from *B. subtilis* str. 168 genomic DNA using the primer pair KinA-Fwd and KinA-Rev and cloned into the NdeI and XhoI sites of pET15b using the In-Fusion method to give pET15bK (Table S3). His-KinA was expressed in *E. coli* strain BL21(DE3)plysS by first growing the cells at 37°C in LB medium supplemented with 100 µg/ml ampicillin and chloramphenicol 17 µg/ml to OD_600_ = 0.4. His-KinA expression was then induced for 16 h at 16°C with 0.5 mM IPTG. All subsequent protein purification steps were carried out at 4°C. The cells were collected by centrifugation, lysed in buffer F (25 mM Tris-HCl (pH 8.0), 450 mM KCl, 5 mM MgCl_2_, 1 mM DTT, 0.1 mM EDTA, and 10% glycerol) supplemented with 1 µM Pepstatin, 1 µM Leupeptin, 20 µg/ml DNase, and 1 mM PMSF. The supernatant was applied to Ni-NTA agarose equilibrated in buffer F. The column was then washed with buffer F containing 20 mM imidazole. To remove the hexahistidine affinity tag, thrombin was added to 0.3 mg/ml Ni-NTA agarose bed volume in buffer F and incubated overnight. KinA was eluted and passed over clean Ni-NTA resin equilibrated in buffer F to remove any uncleaved His-KinA. KinA was concentrated through a 30 kDa cutoff membrane and stored at –80°C.

### Spo0F Labeling

The Spo0F labeling reaction was performed at 25°C and contained 206 µM Spo0F-His, 20 µM KinA, 0.25 µM [γ-^32^P] ATP, and phosphotransfer buffer (50 mM EPPS (pH 8.5), 20 mM MgCl_2_, 0.1 mM EDTA, and 5% glycerol). After 1 h the reaction was quenched with 7.5 mM cold ATP. Spo0F and Spo0F∼P were obtained at a ratio of 4∶1 as determined by native-PAGE electrophoresis followed by Coomassie staining and quantitation with ImageJ [15],[43].

### In Vitro Phosphatase Assay

The Rap phosphatase assays were performed at 25°C and initiated by adding radiolabeled Spo0F∼P prepared as described above to obtain a final reaction containing: 6.5 µM RapH, or 6.5 µM RapJ, or 26 µM RapF, and 6.0 µM radiolabeled Spo0F∼P, 24 µM Spo0F, 2.85 µM KinA, 13.25 mM Tris pH (8.0), 50 mM EPPS (pH 8.5), 0.1 mM EDTA, 100 mM KCl, 23 mM MgCl_2_, 3 mM DTT, 11.6% glycerol 0.04 µM [γ-^32^P] ATP, and 1 mM ATP. Aliquots were removed at the indicated times, mixed with an equal volume of 2X SDS loading buffer, and stored on ice until they were loaded onto 10%–20% polyacrylamide Tris-Tricine-SDS gels. Following PhosphorImager analysis of the gels, Spo0F∼P levels were quantified using ImageQuant.

### In Vitro Phosphotransfer Assay

To measure RapH inhibition of KinA phosphotransfer to Spo0F, reactions were set up by preincubating 6.4 µM wild-type or mutant RapH with 0.1 µM KinA and 0.04 µM [γ-^32^P] ATP in phosphotransfer buffer for 2 min at 25°C. Spo0F was then added to each reaction. The final concentration of the reaction components were: 6.5 µM wild-type or mutant RapH, 16.6 µM Spo0F, 0.1 µM KinA, 5.4 mM Tris (pH 8.0), 50 mM EPPS (pH 8.5), 0.4 mM Bis-Tris (pH 7.2), 0.1 mM EDTA, 41.7 mM KCl, 21.4 mM MgCl_2_, 1.4 mM DTT, 7.9% glycerol, and 0.04 µM [γ-^32^P] ATP. Aliquots were removed at the indicated times. Spo0F∼P levels were determined by Tris-Tricine-SDS PAGE analysis and quantified as described above.

### Construction of *B. subtilis* P*spoIIG*::*luc* Reporter Strains

The *B. subtilis* IS75 *rapH-phrH* null strain was constructed by first replacing chromosomal *rapH-phrH* with a tetracycline resistance cassette. The primers RapH1 and RapH2 were used to amplify a 1 kb DNA fragment upstream of *rapH* in the IS75 chromosome, while primers RapH3 and RapH4 were used to amplify a 1 kb DNA fragment downstream of *phrH* (Table S3). These PCR products were then ligated to a tetracycline cassette to generate upstream(*rapH*)-Tet-downstream (*phrH*). This product was then PCR amplified using the primers RapH1 and RapH4, purified, and transformed into *B. subtilis* IS75. Double crossover between the chromosome regions homologous to the upstream(*rapH*) and downstream(*phrH*) portions of (*rapH*)-Tet-downstream(*phrH*) replaces the entire *rapH-phrH* operon with tetracyline. The Δ*rapH-phrH*::*tet* strain was named BD5031. The *rapH-phrH* deletion in BD5031 was confirmed by DNA sequencing using primers RapH1seq, RapH2seq, RapHseq5R, Tet_seq1, and Tet_seq2, as well as by Western blotting using anti-RapH rabbit antisera (Table S3 and Figure S4). Chromosomal DNA from BD5031 was isolated and transformed into strain PP533, which contains P*spoIIG*::*luc* inserted by Campbell recombination at the native *spoIIG* promoter, to yield strain BD5035 Δ*rapH-phrH*::*tet*, P*spoIIG*::*luc*. To generate site-directed mutations in the BD5035 *rapH* gene, *rapH* was amplified from wild-type *B. subtilis* by PCR and inserted into the SalI and SphI sites in pDR111 (a kind gift from D. Rudner, Harvard Medical School). The resulting plasmid, pDRH1, was then mutagenized using a common forward primer (FWD AMP) in combination with the appropriate mutagenic primer using the ChangeIT Mutagenesis Kit (USB) (Table S3). DNA sequencing confirmed that the *rapH* plasmids were free of mutations other than those introduced by site-directed mutagenesis. pDRH1-derived plasmids were then transformed into BD5035 Δ*rapH-phrH*::*tet*, P*spoIIG*::*luc*, which by double-crossover recombination at the *amyE* locus yields strains expressing the entire wild-type or mutant *rapH* locus under the control of the IPTG-inducible hyperspank promoter.

### Luciferase Bioassays

Luciferase from *Photinus pyralis* was employed as a reporter because it is highly unstable in *B. subtilis*. We detected less than 50% luciferase activity after less than 8 min following the addition of the protein translation inhibitor puromycin to growing bioluminescence reporter strains (unpublished data). Thus, the response dynamics of the assay are such that we are measuring the rate of gene transcription in near real-time rather than the prolonged accumulation of a highly stable reporter such as β-galactosidase. The reporter strains were grown in LB medium to OD_600_≈2, centrifuged, and resuspended in fresh Sporulation Medium (DSM) [44] to OD_600_ = 2. The cultures were then diluted 20-fold in fresh DSM supplemented with 0.25 mM IPTG, and 200 µl were dispensed per well in duplicate in a 96-well black plate (Corning). 10 µl of luciferin was added to each well at a final concentration of 4.7 mM. The cultures were then incubated at 37°C under agitation in a PerkinElmer Envision 2104 Multilabel Reader. The plate lids were heated to 38°C to avoid condensation. Relative Luminescence Unit (RLU) and OD_600_ were measured at 1.5 min intervals.

### Antibody Production and Immunoblotting

Anti-RapH antiserum was recovered from rabbits injected with purified RapH protein (Lampire Biological Laboratories). *B. subtilis* whole-cell lysates were electrophoresed on SDS-PAGE gels and blotted to PVDF membrane. Immunostaining was performed using anti-RapH rabbit antisera followed by anti-rabbit IgG HRP-conjugated antibody. Protein was detected by ECL chemiluminescence (Pharmacia).

### Accession Numbers

Atomic coordinates and structure factors for RapH-Spo0F have been deposited in the Protein Data Bank under accession code 3Q15.

## Supporting Information

Figure S1 The (RapH_B_-Spo0F_B_)_2_ heterotetramer. (A) RapH_B_ (blue), Spo0F_B_ (brown), RapH_B_′ (green), and Spo0F_B_′ (magenta). RapH_B_ residues 27–45 and 292–294 are disordered and represented by blue and green dashed lines in RapH_B_ and RapH_B_′, respectively. Spo0F_B_ residues 83–98 are disordered and are represented by brown and magenta dashed lines, respectively. (B) To obtain this view of RapH_B_, the structure illustrated in panel A was rotated 90° in the direction indicated by the arrow. The RapH_B_ N-terminal 3-helix bundle (light blue) is connected to the C-terminal TPR domain by a flexible linker (black dashed lines) and a short helix (black cylinder). The RapH_B_ disordered residues 27–45 and 292–294 are represented by light blue and grey dashed lines, respectively.
(1.65 MB TIF)

Figure S2 RapH_A_ and RapH_B_ structural alignment. Structural alignment of RapH_A_ (blue) and RapH_B_ (dark pink) illustrates their conformational differences. A portion of the RapH 3-helix bundle (residues 27–45) is disordered in RapH_B_ but ordered in RapH_A_, and residues in and adjacent to this region make regulatory contacts with Spo0F. The N-terminal region of RapH_A_, extending from the 3-helix bundle to TPR1 helix A, is rotated slightly away from the C-terminal TPR domain. The most prominent displacements occur near the C-terminal ends of helices α1 and α3, helix α4, the loop connecting α4 to the N-terminus of TPR1 helix A (α5), and in the residues near the N-terminus of TPR1 helix A. Structural alignments were performed with Dali-Lite [45].
(1.55 MB TIF)

Figure S3 RapH-Spo0F interface. (Bottom panel) Expanded view highlighting the RapH-Spo0F interaction contained within the area enclosed by the black rectangle in the top panel. Spo0F residues 11–18, 21–22, 25, 34–38, 56–60, 83, 84, 104–106, 108, 109, and 112 (magenta) are buried in the RapH (blue) interface. The Spo0F secondary structure elements containing residues buried in the RapH interface are labeled with magenta type. Spo0F residues not buried in the RapH interface are colored yellow. To help illustrate the fact that the RapH-Spo0F interface surrounds the Spo0F active site, the side chain of the Spo0F active-site aspartate-54 is depicted (green sticks).
(2.48 MB TIF)

Figure S4 Western blot analysis of RapH expression. *B. subtilis* whole-cell extracts were analyzed by Western blotting to determine the expression level of wild-type and mutant RapH proteins. Western blotting also confirmed the absence of RapH in strain BD5035 (Δ*rapH-phrH*::*tet*, P*spoIIG*::*luc*).
(0.47 MB TIF)

Table S1Phasing and refinement statistics. Data for the highest resolution shell are given in parentheses. 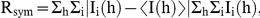 where I_i_(h) is the i^th^ measurement of h and <I(h)> is the mean of all measurements of I(h) for reflection h. 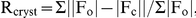 calculated with a working set of reflections. R_free_ is R_cryst_ calculated with only the test set (6.2%) of reflections. The protein molecule average B-factors were calculated using values that included both B-residual and B-TLS. FOM, figure of merit.(0.07 MB DOC)

Table S2 Rap protein amino acid identity in highly conserved positions lying in the RapH-Spo0F interface. RapA, RapB, RapE, RapF, RapH, RapJ, and Rap60 sequences refer to *B. subtilis* Rap proteins. BXA0205 and BA3790 sequences refer to *B. anthracis* Rap proteins. Sequences were aligned in Geneious Pro and analyzed using the ConSurf server [46]. Rap60 is included in this table because previous in vivo studies suggest that Rap60 is a phosphatase, and our alignments show that the Rap60 amino acid sequence conserves all of the residues found to be functionally important for Spo0F dephosphorylation [47]. RapF residue His50 disables its phosphatase activity (H shown in bold).
(0.04 MB DOC)

Table S3 Oligonucleotides.
(0.07 MB DOC)

## Abbreviations

HPt: histidine phosphotransfer
TPR: tetratricopeptide repeat
