# Supplementary figures and images for: Structural Basis of Response Regulator Dephosphorylation by Rap Phosphatases
Source: PLoS Biol. 2011 Feb 8;9(2):e1000589. doi: 10.1371/journal.pbio.1000589 (PMC3035606; doi:10.1371/journal.pbio.1000589)

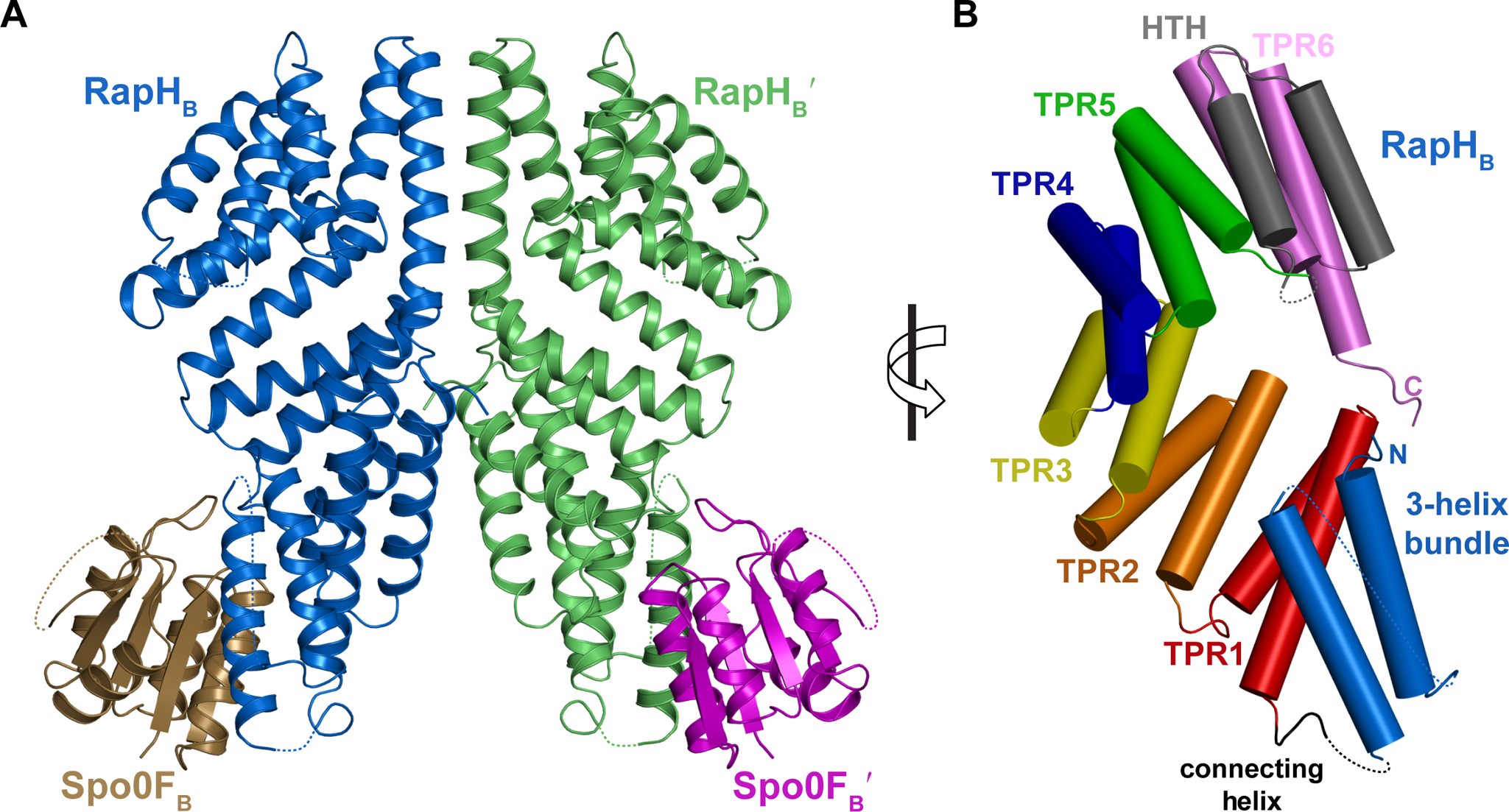

Supplement: Figure S1 — The (RapHB-Spo0FB)2 heterotetramer. (A) RapHB (blue), Spo0FB (brown), RapHB′ (green), and Spo0FB′ (magenta). RapHB residues 27–45 and 292–294 are disordered and represented by blue and green dashed lines in RapHB and RapHB′, respectively. Spo0FB residues 83–98 are disordered and are represented by brown and magenta dashed lines, respectively. (B) To obtain this view of RapHB, the structure illustrated in panel A was rotated 90° in the direction indicated by the arrow. The RapHB N-terminal 3-helix bundle (light blue) is connected to the C-terminal TPR domain by a flexible linker (black dashed lines) and a short helix (black cylinder). The RapHB disordered residues 27–45 and 292–294 are represented by light blue and grey dashed lines, respectively. (1.65 MB TIF) [file pbio.1000589.s001.tif]

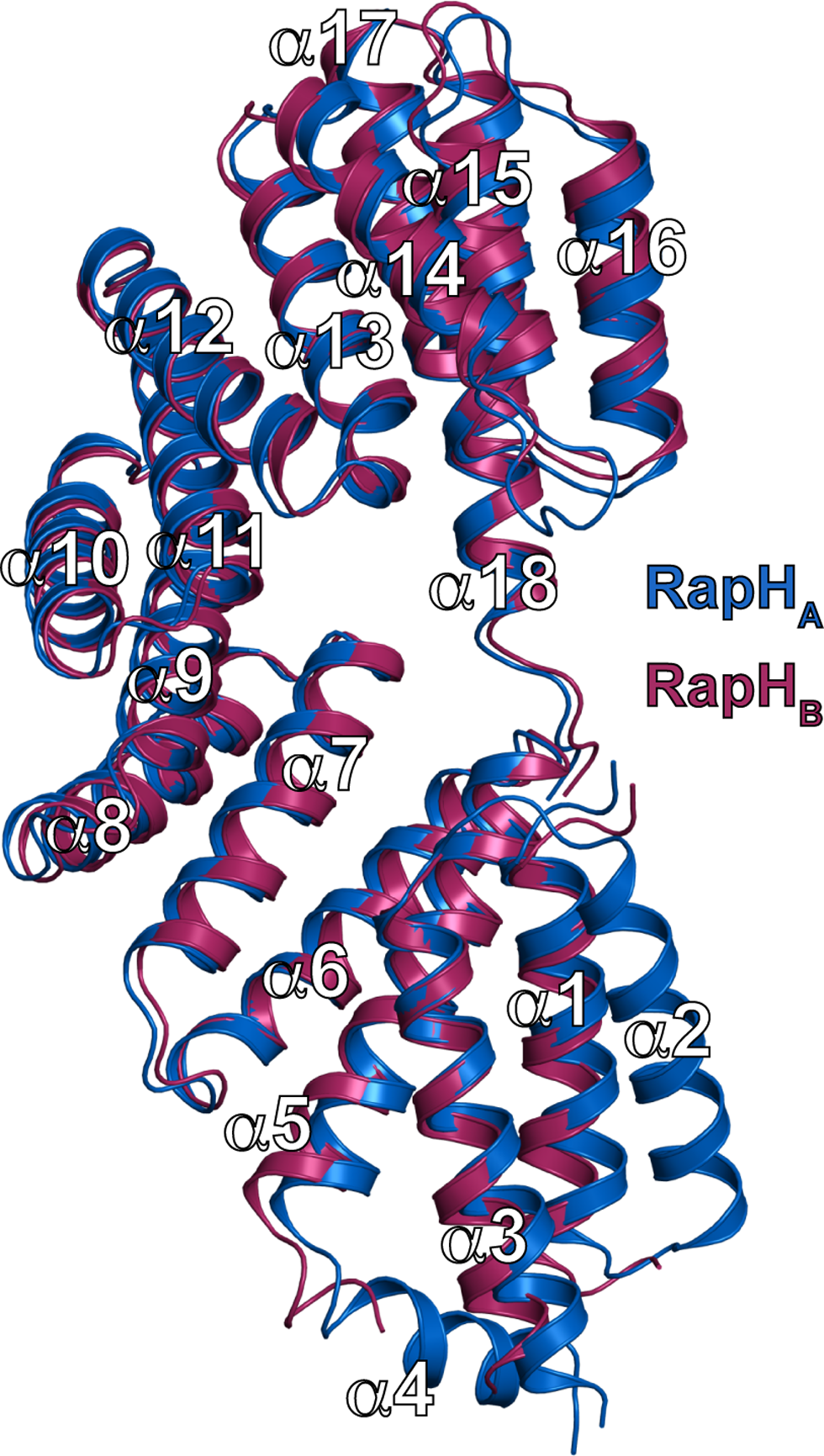

Supplement: Figure S2 — RapHA and RapHB structural alignment. Structural alignment of RapHA (blue) and RapHB (dark pink) illustrates their conformational differences. A portion of the RapH 3-helix bundle (residues 27–45) is disordered in RapHB but ordered in RapHA, and residues in and adjacent to this region make regulatory contacts with Spo0F. The N-terminal region of RapHA, extending from the 3-helix bundle to TPR1 helix A, is rotated slightly away from the C-terminal TPR domain. The most prominent displacements occur near the C-terminal ends of helices α1 and α3, helix α4, the loop connecting α4 to the N-terminus of TPR1 helix A (α5), and in the residues near the N-terminus of TPR1 helix A. Structural alignments were performed with Dali-Lite [45]. (1.55 MB TIF) [file pbio.1000589.s002.tif]

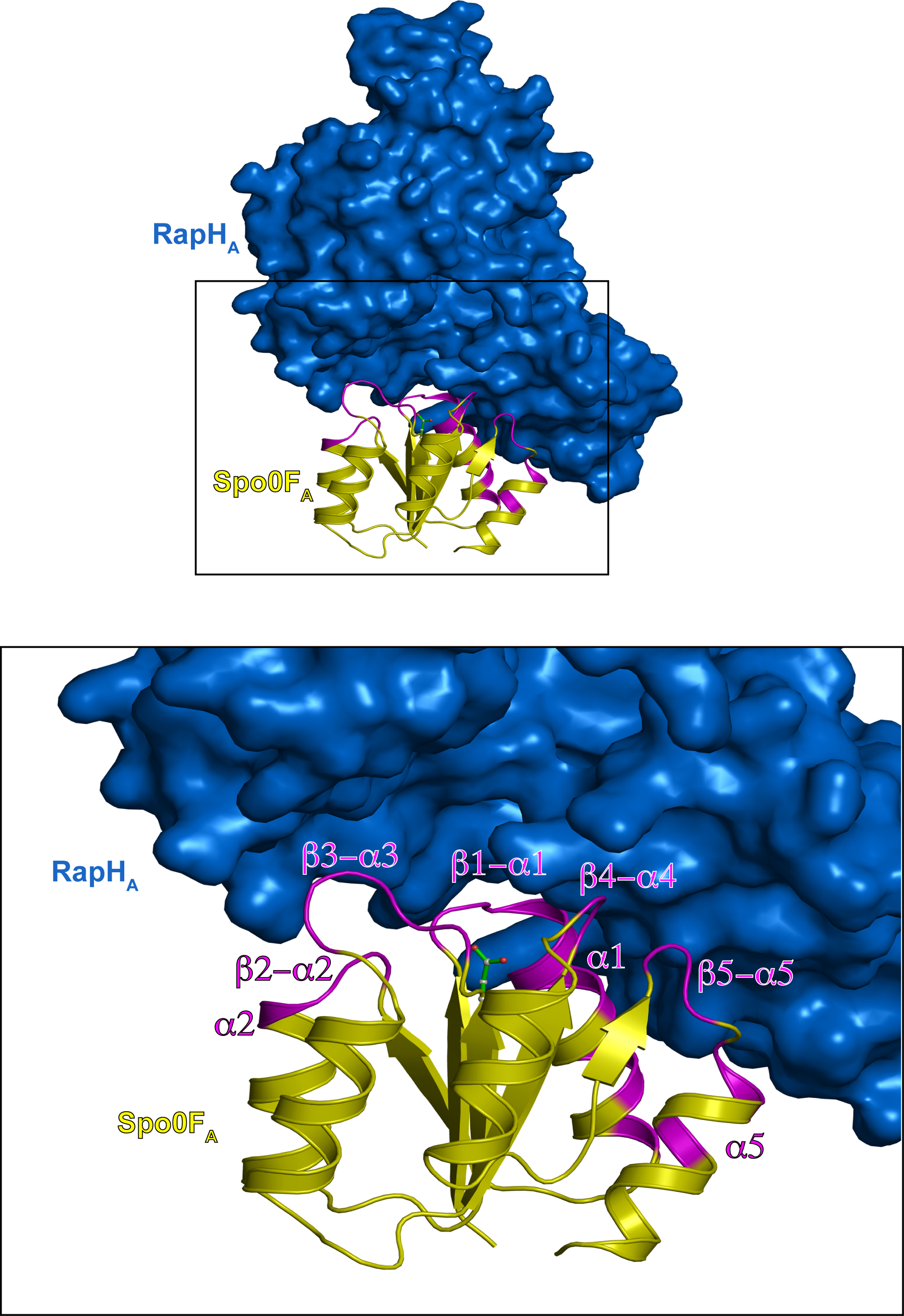

Supplement: Figure S3 — RapH-Spo0F interface. (Bottom panel) Expanded view highlighting the RapH-Spo0F interaction contained within the area enclosed by the black rectangle in the top panel. Spo0F residues 11–18, 21–22, 25, 34–38, 56–60, 83, 84, 104–106, 108, 109, and 112 (magenta) are buried in the RapH (blue) interface. The Spo0F secondary structure elements containing residues buried in the RapH interface are labeled with magenta type. Spo0F residues not buried in the RapH interface are colored yellow. To help illustrate the fact that the RapH-Spo0F interface surrounds the Spo0F active site, the side chain of the Spo0F active-site aspartate-54 is depicted (green sticks). (2.48 MB TIF) [file pbio.1000589.s003.tif]

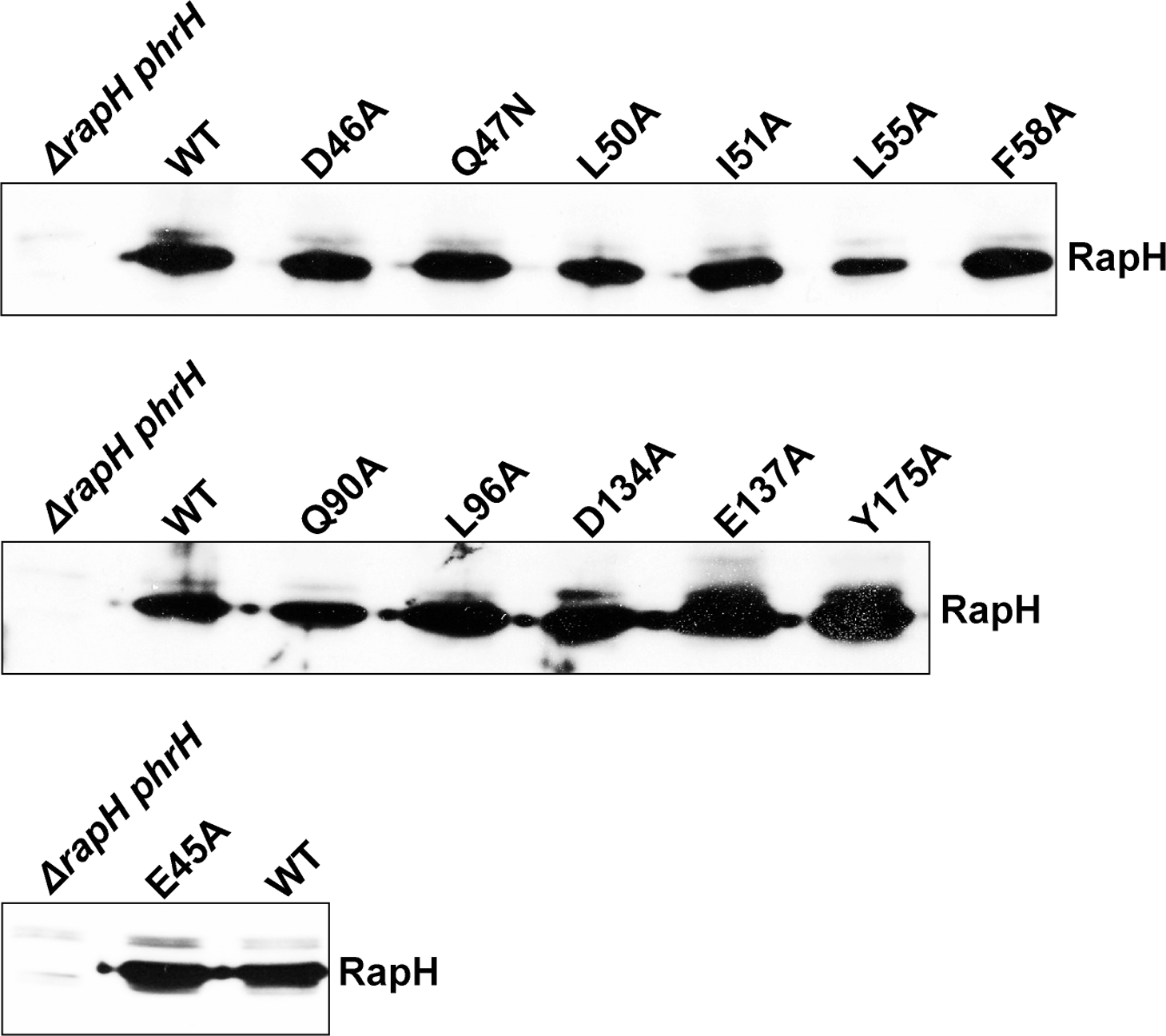

Supplement: Figure S4 — Western blot analysis of RapH expression. B. subtilis whole-cell extracts were analyzed by Western blotting to determine the expression level of wild-type and mutant RapH proteins. Western blotting also confirmed the absence of RapH in strain BD5035 (ΔrapH-phrH::tet, PspoIIG::luc). (0.47 MB TIF) [file pbio.1000589.s004.tif]
