# Supplementary material for: Structural Basis of Response Regulator Dephosphorylation by Rap Phosphatases
Source: PLoS Biol. 2011 Feb 8;9(2):e1000589. doi: 10.1371/journal.pbio.1000589 (PMC3035606; doi:10.1371/journal.pbio.1000589)
Supplement: Table S1 — Phasing and refinement statistics. Data for the highest resolution shell are given in parentheses. where Ii(h) is the ith measurement of h and <I(h)> is the mean of all measurements of I(h) for reflection h. calculated with a working set of reflections. Rfree is Rcryst calculated with only the test set (6.2%) of reflections. The protein molecule average B-factors were calculated using values that included both B-residual and B-TLS. FOM, figure of merit. (0.07 MB DOC) [file pbio.1000589.s005.doc]

|  | **Native** | **SeMet SAD** |
| --- | --- | --- |
|  | RapH-Spo0F | RapH-Spo0F(D54E) |
| **Data collection** |  |  |
| Space group | C2 | C2 |
| Cell dimensions |  |  |
| a, b, c (Å) | 95.06, 157.45, 84.72 | 95.83, 156.58, 84.72 |
| α, β, γ (°) | 90.0, 110.6, 90.0 | 90.0, 110.7, 90.0 |
| Resolution (Å) | 50.00-2.20 (2.24-2.20) | 50.00-2.70 (2.80-2.70) |
| Wavelength (Å) | 1.0750 | 0.9792 |
| Completeness (%) | 98.3 (99.8) | 98.6 (95.7) |
| R_sym_ (%) | 6.3 (51.7) | 8.5 (45.2) |
| Average I / σI | 16.13 (1.80) | 21.07(2.99) |
| Redundancy | 3.69 | 7.52 |
| Total reflections | 215758 | 238,967 |
| Unique reflections | 58465 | 31,782 |
|  |  |  |
| **SAD Phasing** |  |  |
| FOM |  | 0.34 |
|  |  |  |
| **Refinement** |  |  |
| R_work_ / R_free_ (%) | 22.03 (30.28)/26.83 (33.53) |  |
|  |  |  |
| **Number of atoms** |  |  |
| All atoms | 7781 |  |
| RapH_A_ | 3021 |  |
| RapH_B_ | 2755 |  |
| Spo0F_A_ | 933 |  |
| Spo0F_B_ | 812 |  |
| Water | 236 |  |
| Mg^2+^ | 2 |  |
| Glycerol | 12 |  |
| Sulfate | 10 |  |
| **Average B-factor (Å^2^)** |  |  |
| All atoms | 65.17 |  |
| RapH_A_ | 54.54 |  |
| RapH_B_ | 64.54 |  |
| Spo0F_A_ | 84.79 |  |
| Spo0F_B_ | 87.23 |  |
| Water | 54.82 |  |
| Mg2+ | 73.43 |  |
| Glycerol | 38.92 |  |
| Sulfate | 106.88 |  |
| Wilson | 41.45 |  |
| **R.m.s. deviations** |  |  |
| Bond lengths (Å) | 0.004 |  |
| Bond angles (°) | 0.548 |  |
| **Ramachandran statistics** |  |  |
| Favored (%) | 97.54 |  |
| Allowed (%) | 2.46 |  |
| Outliers (%) | 0 |  |
