# Supplementary material for: Structural Basis of Response Regulator Dephosphorylation by Rap Phosphatases
Source: PLoS Biol. 2011 Feb 8;9(2):e1000589. doi: 10.1371/journal.pbio.1000589 (PMC3035606; doi:10.1371/journal.pbio.1000589)
Supplement: Table S2 — Rap protein amino acid identity in highly conserved positions lying in the RapH-Spo0F interface. RapA, RapB, RapE, RapF, RapH, RapJ, and Rap60 sequences refer to B. subtilis Rap proteins. BXA0205 and BA3790 sequences refer to B. anthracis Rap proteins. Sequences were aligned in Geneious Pro and analyzed using the ConSurf server [46]. Rap60 is included in this table because previous in vivo studies suggest that Rap60 is a phosphatase, and our alignments show that the Rap60 amino acid sequence conserves all of the residues found to be functionally important for Spo0F dephosphorylation [47]. RapF residue His50 disables its phosphatase activity (H shown in bold). (0.04 MB DOC) [file pbio.1000589.s006.doc]

| RapH | RapA, RapB, RapE, RapJ, BXA0205, BA3790 | RapF | Rap60 |
| --- | --- | --- | --- |
| L40 | I, I, I, L, I, I | L | I |
| E45 | E, E, E, E, E, Q | E | E |
| D46 | D, D, D, N, D, D | D | N |
| Q47 | Q, Q, Q, Q, Q, Q | Q | Q |
| D48 | D, L, D, D, N, N | D | D |
| L50 | L, L, L, L, L, L | **H** | L |
| I51 | L, L, I, L, L, L | L | I |
| Y53 | Y, Y, Y, F, Y, Y | Y | Y |
| L55 | L, L, L, L, L, L | L | L |
| F58 | F, Y, F, F, F, F | F | F |
| D65 | D, D, E, D, D, D | E | D |
| L96 | L, L, M, L, F, F | L | M |
| V132 | V, V, I, V, I, I | V | V |
| S133 | S, E, S, E, P, P | K | L |
| D134 | D, D, D, D, D, D | D | D |
| I136 | I, I, I, I, I, I | I | I |
| E137 | E, E, E, E, E, E | E | E |
| Y175 | Y, Y, Y, Y, Y, Y | Y | Y |
