# Supplementary material for: Structural Basis of Response Regulator Dephosphorylation by Rap Phosphatases
Source: PLoS Biol. 2011 Feb 8;9(2):e1000589. doi: 10.1371/journal.pbio.1000589 (PMC3035606; doi:10.1371/journal.pbio.1000589)
Supplement: Table S3 — Oligonucleotides. (0.07 MB DOC) [file pbio.1000589.s007.doc]

| **Name** | **Sequence** |
| --- | --- |
| RapH-BamHI | 5′-CGCGGATCCATGAGTCAAGCCATACCGTC-3′ |
| RapH-NotI | 5′-CGTCGCGGCCGCTTAATAGGCATATAAACACTCTCC-3′ |
| Spo0F-BamHI | 5′-GCGGATCCATGATGAATGAAAAAATTTTAATCG-3′ |
| Spo0F-NotI | 5′-CATAGCGGCCGCTCAGTTAGACTTCAGGGGC-3′ |
| Spo0F-NdeI | 5′-GCCATATGATGAATGAAAAAATTT-3′ |
| Spo0F-XhoI | 5′-TATCTCGAGGTTAGACTTCAGG-3′ |
| D54E_Spo0F_T | 5′-CGGCCCGACCTTGTGCTGTTGGAAATGAAAATTCCC-3′ |
| D54E_Spo0F_B | 5′-GGGAATTTTCATTTCCAACAGCACAAGGTCGGGCCG-3′ |
| KinA-Fwd | 5′-CGCGCGGCAGCCATATGGAACAGGATACGCAG-3′ |
| KinA-Rev | 5′-CAGCCGGATCCTCGAGTTATTTTTTTGGAAATGA-3′ |
| RapJ-Fwd | 5′-GAGAACAGATTGGTGGTATGAGAGCAAAGATTCCATCAG-3′ |
| RapJ-Rev | 5′-CAGTCACCCGGGCTCGAGCTATTGAAAACGCTGCTCGG-3′ |
| RapH1 | 5′-CGACGAGAATTCAACCTCTACAGGCGAACGGTTTAACAGG-3′ |
| RapH2 | 5′-CGACGAGGATCCCGGCTTCCCTCCTTCTCTATGAGATATGTCATATTCTA-3′ |
| RapH3 | 5′-CGACGAAAGCTTGGCTTTTTCTTGCTTTACGGAAGACGGTTC-3′ |
| RapH4 | 5′-CGACGATCTAGACTTATAGATCGTATAGGCGGCCGCCTACATAC-3′ |
| RapH1seq | 5′-CCATGTCCCTTTTTCAATCACAGTC-3′ |
| RapH2seq | 5′-GGCGTTCGGGAAAAACGGCGGAAAC-3′ |
| Tet_seq1 | 5′-CAAAATTATTGATTAGCTTTATATCAGCC-3′ |
| Tet_seq2 | 5′-GTAATTTGTTATTACTTTTTTCAGGAATC-3′ |
| RapHseq5R | 5′-CTTGTACACGGCCTGCAAGAACAGAAACAA-3′ |
| E45A_RapH | 5′-CAGCAGATGGAAGCAGATCAGGATTTACTG-3′ |
| R123A_RapH | 5′-ATCGGATATTATGCAGAGGCGGAGAAAGAACT-3′ |
| Q154A_RapH | 5′-TATTATCACATGAAGGCAACCCATGTGTCGATG-3′ |
| F193A_RapH | 5′-AACTATGATGATGCCAAACATTATGATAAAGC-3′ |
| E345A_RapH | 5′-TGCCGCTGTTTTTGCAAGCAGCTGTCACTTTG-3′ |
| E361A_RapH | 5′-TTATCGGAAAGTGGCGAAAGCCCAAGAAGATA-3′ |
| Y376A_RapH | 5′-TGTTTATATGCCGCTTATGAGTCGACAGAATTC-3′ |
| D46A_RapH | 5′-ATTCAGCAGATGGAAGAAGCTCAGGATTTACTGA-3′ |
| L50A_RapH | 5′-GATCAGGATTTAGCGATCTATTATTCTCTGAT-3′ |
| I51A_RapH | 5′-AAGATCAGGATTTACTGGCCTATTATTCTCTGA-3′ |
| L55A_RapH | 5′-GATCTATTATTCTGCGATGTGTTTTCGGCACC-3′ |
| F58A_RapH | 5′-ATTATTCTCTGATGTGTGCTCGGCACCAGCTGAT-3′ |
| Q90A_RapH | 5′-GATCGAGACCCCTGCGAAAAAACTCACAGGT-3′ |
| L96A_RapH | 5′-AAAAACTCACAGGTGCTTTGAAATACTACTC-3′ |
| D134A_RapH | 5′-CCGTTTGTGTCAGCAGATATTGAGAAAGCGGAA-3′ |
| E137A_RapH | 5′-CGTTTGTGTCAGATGATATTGCGAAAGCGGAAT-3′ |
| Y175A_RapH | 5′-AACCATCCTCTAGCCAGCATTAGAACGATACAAAG-3′ |
| Y376A_RapH | 5′-TGTTTATATGCCGCTTAAGTCGACAAGAATTC-3′ |
| Y376A_RapH_Phs1 | 5′-TGTTTATATGCCGCTTAAGCATGCAAGCTAATT-3′ |
| RapH_Q47N_T | 5′-CAGCAGATGGAAGAAGATAACGATTTACTGATC-3′ |
| RapH_Q47N_B | 5′-GATCAGTAAATCGTTATCTTCTTCCATCTGCTG-3′ |
| H50L_RapF | 5′-GAAGATCAAGACCTTCTTTTGTACTATTCACTG-3′ |
